# A comprehensive test set of epoxidation rate constants for iron(iv)–oxo porphyrin cation radical complexes†

**DOI:** 10.1039/c4sc02717e

**Published:** 2014-12-08

**Authors:** Mala A. Sainna, Suresh Kumar, Devesh Kumar, Simonetta Fornarini, Maria Elisa Crestoni, Sam P. de Visser

**Affiliations:** a Manchester Institute of Biotechnology and School of Chemical Engineering and Analytical Science, The University of Manchester 131 Princess Street Manchester M1 7DN UK sam.devisser@manchester.ac.uk; b Department of Applied Physics, School for Physical Sciences, Babasaheb Bhimrao Ambedkar University Vidya Vihar, Rai Bareilly Road Lucknow 226 025 India dkclcre@yahoo.com; c Dipartimento di Chimica e Tecnologie del Farmaco, Università di Roma “La Sapienza” P.le A. Moro 5 00185 Roma Italy mariaelisa.crestoni@uniroma1.it simonetta.fornarini@uniroma1.it

## Abstract

Cytochrome P450 enzymes are heme based monoxygenases that catalyse a range of oxygen atom transfer reactions with various substrates, including aliphatic and aromatic hydroxylation as well as epoxidation reactions. The active species is short-lived and difficult to trap and characterize experimentally, moreover, it reacts in a regioselective manner with substrates leading to aliphatic hydroxylation and epoxidation products, but the origin of this regioselectivity is poorly understood. We have synthesized a model complex and studied it with low-pressure Fourier transform-ion cyclotron resonance (FT-ICR) mass spectrometry (MS). A novel approach was devised using the reaction of [Fe^III^(TPFPP)]^+^ (TPFPP = *meso*-tetrakis(pentafluorophenyl)porphinato dianion) with iodosylbenzene as a terminal oxidant which leads to the production of ions corresponding to [Fe^IV^(O)(TPFPP^+^˙)]^+^. This species was isolated in the gas-phase and studied in its reactivity with a variety of olefins. Product patterns and rate constants under Ideal Gas conditions were determined by FT-ICR MS. All substrates react with [Fe^IV^(O)(TPFPP^+^˙)]^+^ by a more or less efficient oxygen atom transfer process. In addition, substrates with low ionization energies react by a charge-transfer channel, which enabled us to determine the electron affinity of [Fe^IV^(O)(TPFPP^+^˙)]^+^ for the first time. Interestingly, no hydrogen atom abstraction pathways are observed for the reaction of [Fe^IV^(O)(TPFPP^+^˙)]^+^ with prototypical olefins such as propene, cyclohexene and cyclohexadiene and also no kinetic isotope effect in the reaction rate is found, which suggests that the competition between epoxidation and hydroxylation – in the gas-phase – is in favour of substrate epoxidation. This notion further implies that P450 enzymes will need to adapt their substrate binding pocket, in order to enable favourable aliphatic hydroxylation over double bond epoxidation pathways. The MS studies yield a large test-set of experimental reaction rates of iron(iv)–oxo porphyrin cation radical complexes, so far unprecedented in the gas-phase, providing a benchmark for calibration studies using computational techniques. Preliminary computational results presented here confirm the observed trends excellently and rationalize the reactivities within the framework of thermochemical considerations and valence bond schemes.

## Abstract 2

Trends in oxygen atom transfer to Compound I of the P450 models with an extensive test set have been studied and show a preferred regioselectivity of epoxidation over hydroxylation in the gas-phase for the first time.

## Graphical abstract

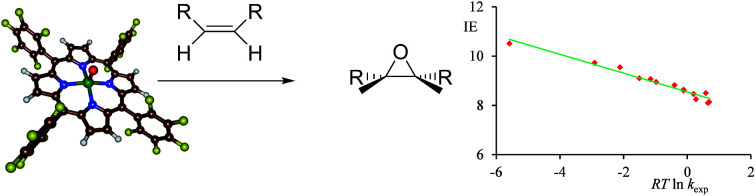

## Introduction

The cytochromes P450 are part of the body's natural defence mechanism in the liver and perform vital functions for human health that include the biodegradation of xenobiotic and drug molecules.^1^ Due to this broad chemical function the P450s can bind and activate a large range of substrates with varying shapes and sizes. Generally, the P450s act as monoxygenases, whereby they bind and utilize molecular oxygen *via* a heme centre and transfer one of the oxygen atoms of O_2_ to a substrate, while the second oxygen atom leaves the process as a water molecule. The P450s react with substrates activating aliphatic and aromatic hydroxylation, epoxidation and sulfoxidation reactions, but have also been reported to catalyse desaturation and *N*-dealkylation reactions.^2^ There are many different P450 isozymes and until early 2014 thousands of different structures had been characterized.^3^ All P450s share common features which include a catalytically active heme group with a central iron atom that is linked to the protein by the thiolate sulphur atom of a cysteinate side chain.^4^

**Fig. 1:**
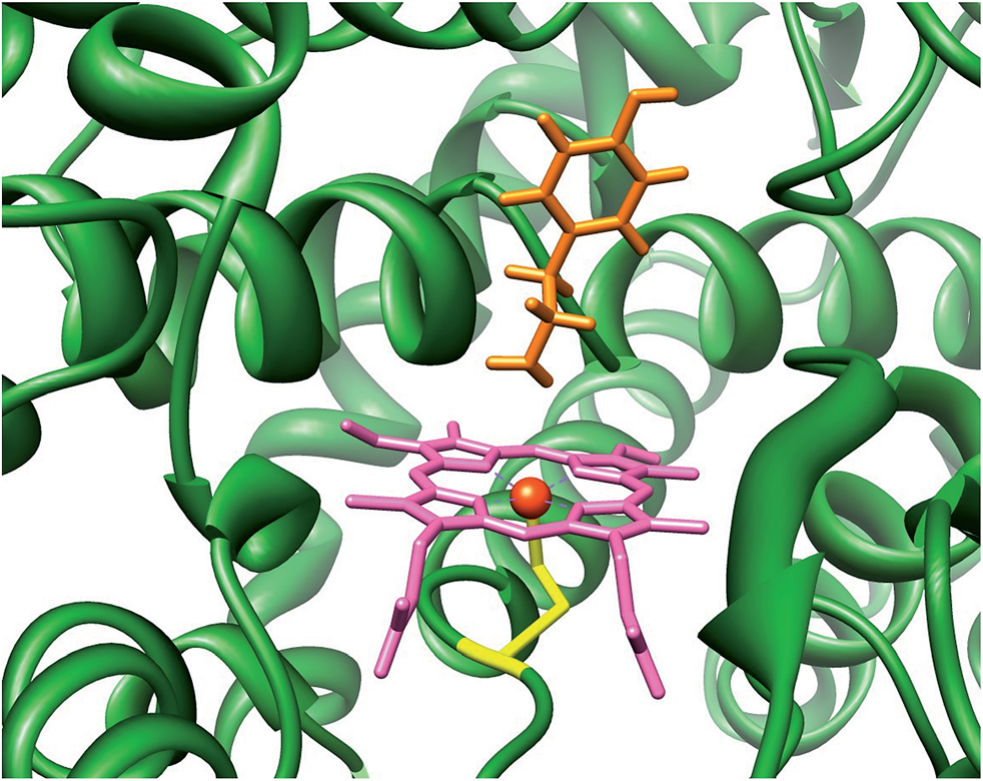
Active site of P450 as taken from the 2WM4 pdb file. Substrate tyramine is highlighted in orange.

Fig. 1 displays the structure of a typical P450 active site, namely the one belonging to the CYP124 isozyme as taken from the 2WM4 protein databank (pdb) file.^5^ As shown in Fig. 1 the substrate (tyramine) is located in a cleft nearby the heme, the substrate binding pocket, which is in a tight orientation with stabilizing hydrogen bonding interactions by several residues. The vacant sixth coordination site of iron is the position where molecular oxygen will bind during the catalytic cycle. The process includes two reduction and two protonation steps to synthesize the active species of P450 called Compound I (Cpd I).^6^ Cpd I is highly reactive and therefore difficult to study experimentally, however, a few reports on its spectroscopic properties have appeared in the literature.^7^

Due to the short lifetime of Cpd I, studying catalytic mechanisms and reaction rates of P450 catalysed reactions is challenging;^8^ therefore, many studies have focused on biomimetic iron–porphyrin complexes instead.^9^ These studies gave detailed insight into the effect of axial and equatorial ligands,^10^ but also on the local environment such as the substrate binding pocket. A particularly useful method to establish the properties and reactivity patterns of short-lived complexes, such as catalytic intermediates, is Fourier transform – ion cyclotron resonance (FT-ICR) mass spectrometry (MS).^11^ In FT-ICR MS, the charged species of interest (either positive or negative ion) is trapped in a collision cell for a specific time during which reactions with neutral gases can occur and be studied at the prevailing low pressure of the instrument. FT-ICR MS allows one to measure the ion distributions and fragmentation patterns at varying trapping time, thereby yielding insight into reactivities, and enabling one to calculate rate constants and thermochemical properties. In recent work, Crestoni, Fornarini and co-workers have succeeded in trapping and characterizing the Cpd I analogues of iron and manganese porphyrin complexes and studied their reactivity with a selection of substrates.^12^ Thus, the [Mn^V^(O)(TPFPP)]^+^ complex (TPFPP = *meso*-tetrakis (pentafluorophenyl)porphinato dianion) was found to react with model substrates through oxygen atom transfer (OAT), electron transfer (ET), hydride transfer and ligand addition. However, no direct hydrogen atom transfer (HAT) with any tested substrate took place.

In order to find out what drives the OAT reaction of Cpd I with olefins, we decided to investigate the properties and reactivities of [Fe^IV^(O)(Por^+^˙)]^+^ (Por = porphine dianion) and [Fe^IV^(O)(TPFPP^+^˙)]^+^ with FT-ICR MS and with density functional theory (DFT) methods. These studies represent the first comprehensive – experimental and computational – study on olefin epoxidation by iron(iv)–oxo porphyrin cation radical models and allow correlations to be established between the OAT rate constant and the ionization energy (IE) of the olefin. These correlations are further supported and rationalized by computational modelling.

## Methods

### Materials

All chemicals used in the experiments, including (5,10,15,20-tetrakis(pentafluorophenyl)porphinato)iron(iii) chloride, [Fe^III^(TPFPP)]Cl, were research grade products purchased from commercial sources and used as received. All the solvents were analytical grade. Ethylene, propylene, and *E*-2-butene were high purity gases from Matheson Gas Products Inc. Iodosylbenzene (C_6_H_5_IO) was prepared according to a published procedure and stored at −20 °C.^13^

### Instrumental

All experiments were run on a Bruker BioApex Fourier transform-ion cyclotron resonance (FT-ICR) mass spectrometer equipped with an Apollo I electrospray ionization source, a 4.7 T superconducting magnet, and a cylindrical infinity cell. Analyte solutions were continuously infused through a 50 μm internal diameter fused-silica capillary at a flow rate of 120 μL h^−1^ by a syringe pump and ions were accumulated in a radiofrequency-only hexapole ion guide for 0.8 s. The ion population, desolvated by a heated (380 K) N_2_ counter current drying gas, was pulsed into the ICR cell at room temperature (300 K), where the ion of interest was isolated by ion ejection procedures and allowed to react with the selected neutral reagent (L) admitted by a needle valve at stationary pressures in the range of 1.0–15 × 10^−8^ mbar. The pressure was measured with a cold-cathode sensor (IKR Pfeiffer Balzers S.p.A., Milan, Italy) calibrated by using the rate constant, *k*_methane_ = 1.1 × 10^−9^ cm^3^ s^−1^, for the reference reaction CH_4_^+^˙ + CH_4_ → CH_5_^+^ + CH_3_˙ and corrected for different response factors.^14^

Pseudo-first order rate constants were obtained from the slope of the semi-logarithmic decrease of the parent ion abundance as a function of time and divided by the substrate concentration to determine the second-order rate constants (*k*_exp_) at 300 K. The reaction efficiencies (*Φ*) are percentages of the second-order rate constant with respect to the collision rate constant (*k*_ADO_), *i.e. Φ* = *k*_exp_/*k*_ADO_, calculated by the parametrized trajectory theory.^15^ These values and product ion branching ratios were found to be independent of the pressure of the neutral and of the additional presence of an inert bath gas, *i.e.* argon. The reproducibility of the *k*_exp_ values was within 10%, while the error of the absolute rate constants is estimated to be ±30%.

### Sample preparation

The [Fe^IV^(O)(TPFPP^+^˙)]^+^ ion of interest was prepared by adding iodosylbenzene (0.5 mM) to [Fe^III^(TPFPP)]Cl (10 μM) in a CH_3_OH/CH_2_Cl_2_ (1 : 1) solvent mixture cooled at −40 °C. The so-formed [Fe^IV^(O)(TPFPP^+^˙)]^+^ ion persisted at this temperature for about 1 h. The high-resolution electrospray ionization FT-ICR mass analysis shows the presence of ions [Fe^IV^(O)(TPFPP^+^˙)]^+^ as a prominent cluster centered at *m*/*z* 1044, along with the resting form [Fe^III^(TPFPP)]^+^, characterized by the same isotopic pattern, and centered at *m*/*z* 1028.

As already pointed out,^16^ the synthetic method yields a portion of an isomeric species, likely oxidized on the porphyrin ligand and ineffective in any OAT reaction to reductants. This OAT-unreactive fraction of the overall ion population at *m*/*z* 1044 has been identified as a four-coordinate iron(iii) complex incorporating an O-atom on the porphyrin ligand, henceforth denoted as [Fe^III^(TPFPP-O)]^+^.^16^ Its relative amount has been evaluated exploiting the characteristic reaction with NO, displaying different paths from the two isomeric species. In fact, while [Fe^III^(TPFPP-O)]^+^ yields a ligand addition product, [Fe^III^(TPFPP-O)(NO)]^+^, the genuine iron(iv)–oxo complex [Fe^IV^(O)(TPFPP^+^˙)]^+^ undergoes an OAT process by releasing [Fe^III^(TPFPP)]^+^.

### Computation

**Scheme 1:**
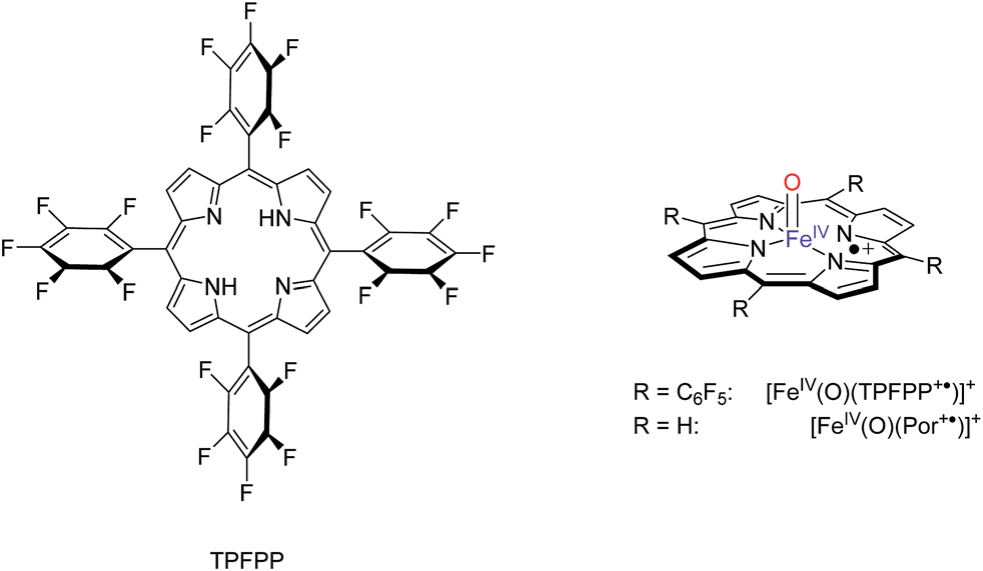
Models investigated in this work.

All calculations discussed here utilize density functional theory (DFT) methods as implemented in the Jaguar and Gaussian-09 program packages.^17^ Two different models were investigated: (i) [Fe^IV^(O)(Por^+^˙)]^+^ (A) that includes a porphyrin (Por) ring with all side-chains abbreviated to hydrogen atoms, and (ii) [Fe^IV^(O)(TPFPP^+^˙)]^+^ (B), Scheme 1. Similar to previous work of ours in the field,^18^ we use the unrestricted hybrid density functional method UB3LYP^19^ as it was shown to reproduce the kinetics of metal(iv)–oxo oxidants well.^20^ Initial exploratory calculations employed a modest LANL2DZ basis set on iron and 6-31G on the rest of the atoms (basis set BS1)^21^ for geometry optimizations, analytical frequencies and geometry scans. These studies explored the potential energy surfaces involving reactants, intermediates and products on different spin states in detail and generated starting structures for the transition state optimizations. All local minima reported here had real frequencies only and the transition states were characterized by a single imaginary frequency for the correct mode. To improve the energetics of these structures we did single point calculations in the gas-phase with a triple-ζ quality basis set on iron (LACV3P+) and 6-311+G* on the rest of the atoms, basis set BS2. Subsequently, all geometries (local minima and transition states) were reoptimized at the UB3LYP/BS2 and UB3LYP-D3/BS2 levels of theory^19,22^ and characterized by an analytical frequency analysis. Barrier heights reported in this work were calculated relative to isolated reactants, although using reactant complexes instead only minor changes are observed (ESI†).

The effect of solvent on the rate constants was tested through single point calculations using the self-consistent reactant field model as implemented in Gaussian with a dielectric constant representing chloroform (*ε* = 4.7113).

Ionization energies and bond dissociation energy (BDE_OH_) values were calculated as before^23^ and represent adiabatic values for reaction 1 and 2, respectively and report UB3LYP/BS2//UB3LYP/BS1 energies including ZPE and dispersion corrections.

1 A → A^+^˙ + e^−^ + IE_A_

2 [Fe^IV^(OH)(Por)]^+^ → [Fe^IV^(O)(Por^+^˙)]^+^ + H˙ + BDE_OH_

## Results

### Gas phase reactivity with FT-ICR MS

#### Formation and characterization of naked [Fe^IV^(O)(TPFPP^+^˙)]^+^ ions

In an early survey, the preparation of a high-valent iron(iv)–oxo porphyrin cation radical was achieved by controlled oxidation of [Fe^III^(TPFPP)]Cl with H_2_O_2_ in a methanol solution. This solution was then sampled by electrospray ionization FT-ICR MS.^12*a*^ However, under the selected experimental conditions, in addition to the heterolytic cleavage of the peroxide bond also a homolytic pathway is observed that yields a high-valent iron(iv)–hydroxo porphyrin complex [Fe^IV^(OH)(TPFPP)]^+^. Because the latter complex differs by one mass unit only from the species of interest, this presence complicates the ion distribution and the assignment of the oxidant in the reaction mixture. Therefore, we aimed to synthesize [Fe^IV^(O)(TPFPP^+^˙)]^+^ through an alternative, neater mechanism. A methanol/dichloromethane solution of [Fe^III^(TPFPP)]Cl was treated with iodosylbenzene (PhIO) as oxygen atom donor. The reaction mixture, assayed by electrospray ionization and high resolution mass measurements by FT-ICR mass spectrometry, displays an ion cluster centered at *m*/*z* 1044.0116, which corresponds to the acquisition of just one oxygen atom by the reactant species [Fe^III^(TPFPP)]^+^.

The thus generated, naked five-coordinate species proves to be resistant with respect to any unimolecular dissociation process (*k*_diss_ ≤ 0.001 s^−1^). However, when the ion-molecule reactivity of the ion cluster at *m*/*z* 1044 is examined through the addition of NO into the FT-ICR cell, two reaction products are observed: (i) a major fraction of ions reacts by OAT and forms [Fe^III^(TPFPP)]^+^ and NO_2_ as the likely neutral product, (ii) a significant percentage of the starting ions undergo a ligand addition reaction to give a product with *m*/*z* 1074.

The product formed in the latter pathway has been sampled by collision induced dissociation (CID), and displays the loss of NO as unique fragmentation channel thus suggesting that NO has become part of the complex as intact ligand at a formerly vacant axial position. Note here that ligand addition is typical for the reactivity of tetracoordinate [Fe^III^(TPFPP)]^+^ ions.^12*a*^ This finding provides circumstantial evidence for an ion population of the same elemental composition as [Fe^IV^(O)(TPFPP^+^˙)]^+^, that may correspond to an isomeric structure oxidized on the porphyrin ligand, designated as [Fe^III^(TPFPP-O)]^+^.

The chemical titration reaction with NO is a way to resolve the isomeric distribution of electrosprayed ions at *m*/*z* 1044 and allowed us to establish the relative amount of [Fe^IV^(O)(TPFPP^+^˙)]^+^ in each experiment, which is consistently found to be equal to or larger than 60% of the total ion population at *m*/*z* 1044.

#### Reactivity of naked [Fe^IV^(O)(TPFPP^+^˙)]^+^ ions with olefins

**Scheme 2:**
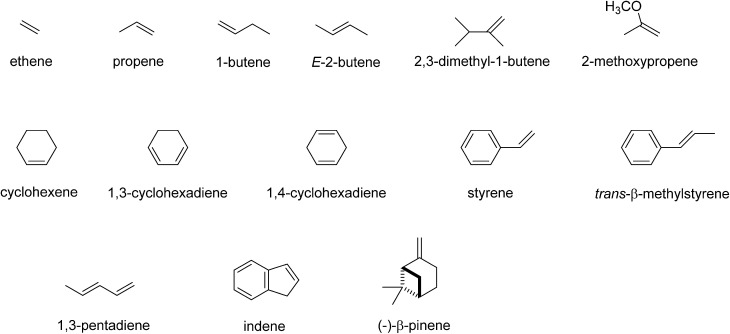
Substrates investigated in this work.

**Scheme 3:**
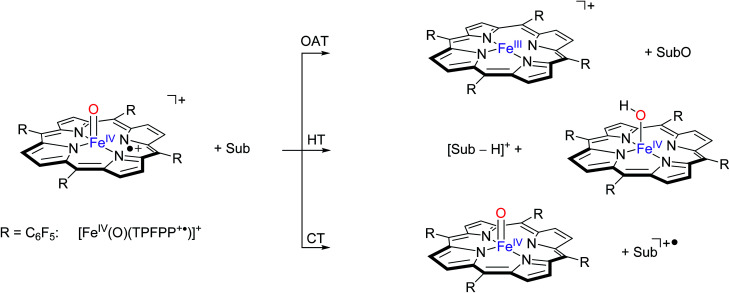
Pathways observed for the reaction of [Fe^IV^(O)(TPFPP^+^˙)]^+^ ions (R = C_6_F_5_) with selected substrates (Sub) as studied with FT-ICR MS.

Our initial studies focused on experimentally determining the reactivity of [Fe^IV^(O)(TPFPP^+^˙)]^+^ with the selected olefins depicted in Scheme 2, and the results are summarized in Table 1. These olefins vary in molecular size and structure and their reported ionization energies span from 8.1 to 10.51 eV.^24^ The [Fe^IV^(O)(TPFPP^+^˙)]^+^ ion was prepared from the reaction of iodosylbenzene with [Fe^III^(TPFPP)]Cl in methanol/dichloromethane solution and transferred by electrospray ionization into the mass spectrometer as described above. Subsequently, the [Fe^IV^(O)(TPFPP^+^˙)]^+^ ion was mass selected and trapped in the FT-ICR cell. The chosen olefin (Sub) was present at constant pressure in the cell and mass spectra were recorded at regular time intervals after isolation of the parent ion. Over time, all [Fe^IV^(O)(TPFPP^+^˙)]^+^ was found to react and new peaks corresponding to product ions appeared in the spectrum. Three different reaction paths were observed and monitored, namely oxygen atom transfer (OAT), hydride transfer (HT) and charge transfer (CT), as illustrated in Scheme 3.

**Table 1:** Kinetic data and product distributions obtained for the gas phase reaction of [Fe^IV^(O)(TPFPP^+^˙)]^+^ with selected olefins as determined by FT-ICR MS

Substrate	IE^a^	*k*_exp_ b	*k*_ADO_ c	*Φ* d	HT	CT	OAT	Add
Ethene	10.51	8.5 × 10^−5^	8.5	1 × 10^−3^	—	—	100	—
Propene	9.73	7.6 × 10^−3^	9.45	0.080	—	—	100	—
1-Butene	9.55	0.029	9.6	0.30	—	—	100	—
*E*-2-Butene	9.10	0.080	10.8	0.74	—	—	100	—
2,3-Dimethyl-1-butene	9.07	0.145	9.5	1.5	—	—	100	—
Cyclohexene^e^	8.95	0.194–0.291	9.7	2–3	—	—	75	25
1,4-Cyclohexadiene	8.82	0.511	9.29	5.5	—	—	90	10
2-Methoxy-1-propene	8.64	0.819	10.5	7.8	—	—	100	—
1,3-Pentadiene	8.60	0.826	9.6	8.6	—	—	100	—
Styrene	8.46	1.40	9.26	15	—	—	100	—
1,3-Cyclohexadiene	8.25	1.58	9.29	17	—	—	100	—
*trans*-β-Methylstyrene	8.1–8.2	2.97	11.9	25	4	—	96	—
Indene	8.14	3.18	8.6	37	2	12	86	—
β-Pinene^f^	N/A	4.32–4.7	9.4	46–50	—	—	100	—

a Ionization energies (IE, eV) are from ref. 24. N/A stands for not available.

b Second-order rate constants (*k*_exp_) in units of 10^−10^ cm^3^ molecule^−1^ s^−1^ are measured at a temperature of 300 K in the FT-ICR cell. The estimated error in *k*_exp_ is ±30%, although the internal consistency of the data is within ±10%.

c Collision rate constants (*k*_ADO_) evaluated with the parameterized trajectory theory.

d Reaction efficiency (%), *Φ* = *k*_exp_/*k*_ADO_ × 100.

e The reaction with cyclohexene-*d*_10_ gave a rate constant within experimental error of that for cyclohexene-*h*_10_.

f The IE for α-pinene is 8.07 eV.

These three pathways lead to different product ions in the recorded spectra: (i) [Fe^III^(TPFPP)]^+^ ions are the products of the OAT channel; (ii) ions at *m*/*z* corresponding to [Sub-H]^+^ are obtained from the HT processes; (iii) ions corresponding to Sub^+^˙ arise from a CT reaction. Interestingly, no evidence of hydrogen atom abstraction (HAT) is observed with any of the selected olefins listed in Table 1. In the case of two substrates, cyclohexene and 1,4-cyclohexadiene, molecular addition (Add) to the complex is also observed, leading to an ion with *m*/*z* value formally corresponding to [Fe(O)(TPFPP)(Sub)]^+^. It is possible that these addition complexes are products from a hydrogen atom abstraction reaction but currently this cannot be established from the product ions. However, experiments with fully deuterated cyclohexene (cyclohexene-*d*_10_) *versus* that of cyclohexene-*h*_10_ gave a rate constant ratio *k*_H_/*k*_D_ very close to 1. Reactions starting with an initial hydrogen atom abstraction reaction normally encounter a large kinetic isotope effect (KIE = *k*_H_/*k*_D_) of well greater than 1, hence these KIE experiments implicate that no rate determining hydrogen atom abstraction takes place here.

Because the selected ions at *m*/*z* 1044 comprise isomeric species as described in the previous paragraph, it is important to mention that in all experiments with the selected substrates (olefins and unsaturated hydrocarbons) an unreactive fraction is observed in a corresponding relative amount as the species yielding the NO addition product in the probe reaction with nitric oxide. This species has been assigned the features of a porphyrin-oxidized complex and its contribution to the experiments has been thoroughly subtracted, an operation allowed by the distinctly different (un)reactivity of the two isomeric complexes [Fe^IV^(O)(TPFPP^+^˙)]^+^ and [Fe^III^(TPFPP-O)]^+^. Henceforth, the implication of this species will not be further discussed.

**Fig. 2:**
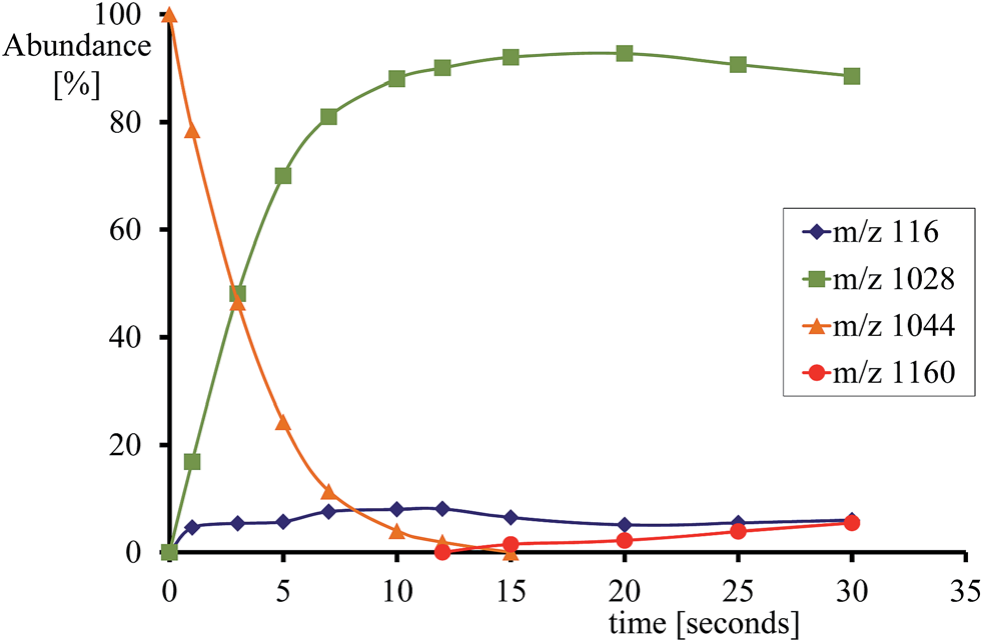
Time dependence of relative ion abundancies for the reaction of [Fe^IV^(O)(TPFPP^+^˙)]^+^ (*m*/*z* 1044) with indene. Product ions are [Fe^III^(TPFPP)]^+^ (*m*/*z* 1028), [Fe(TPFPP)(C_9_H_8_)O]^+^ (*m*/*z* 1160) and C_9_H_8_^+^˙ (*m*/*z* 116). Experiments were performed in the presence of indene at 5.2 × 10^−8^ mbar in the FT-ICR cell.

Fig. 2 gives an example of the time dependence of the relative ion abundance of reactant and product ions as a function of time for the reaction of [Fe^IV^(O)(TPFPP^+^˙)]^+^ with indene. In general, the ion abundance of [Fe^IV^(O)(TPFPP^+^˙)]^+^ follows an exponential decay as a function of time, as illustrated by its reaction with indene in Fig. 2. The pseudo-first order decay of the reactant ion abundance as a function of time was then converted into a bimolecular rate constant, *k*_exp_, which was normalized by the respective collision rate constant (*k*_ADO_) to give the relative efficiency (*Φ* = *k*_exp_/*k*_ADO_ × 100).^15^ The gas phase reactivity of [Fe^IV^(O)(TPFPP^+^˙)]^+^ spans a wide range of reaction efficiencies, with values varying from 0.001% to 0.08% for terminal olefins, like ethene and propene, up to *ca.* 50% for electron-rich monoterpenes.

With most substrates a clean OAT conversion from reactants to products is observed without production of by-products and the time dependence shows a pattern like that displayed in Fig. 2. In the case of cyclohexene, about 25% addition occurs with the formation of an ion formally corresponding to [Fe(TPFPP)(c-C_6_H_10_)O]^+^. The formation of a long-lived complex with this substrate suggests that, within the metal coordination sphere, the olefin is activated to form an O-containing species endowed with appreciable affinity for the metal. Ligand binding to iron porphyrin complexes has been found to correlate with the gas phase basicity of the ligand.^25^ It may thus be inferred that the olefin has turned into a more basic species, either an epoxide by O-addition across the double bond or an alcohol by allylic C–H bond activation. The nature of the adduct complex has been further probed and confirmed by low-energy CID, and reveals the formation of the reduced species [Fe^III^(TPFPP)]^+^ through the release of SubO. The so-formed complex from the reaction of [Fe^IV^(O)(TPFPP^+^˙)]^+^ with the olefin substrate may then be depicted as [Fe^III^(TPFPP)(SubO)]^+^. Similar product complexes have been observed previously from other substrates through oxygen atom-acceptor properties such as sulphides, amines, and phosphorus containing complexes.^12*a*,16^

In addition, substrates with low ionization potential are found to react *via* hydride transfer (*trans*-β-methylstyrene, and indene) and/or charge transfer reactions (indene) as side-reactions. We examined whether a charge-transfer (CT) reaction occurs between [Fe^IV^(O)(TPFPP^+^˙)]^+^ and sampled substrates. As in FT-ICR MS only exothermic reactions are able to occur due to the low number of collisions in the gas, the data in Table 1 show that the charge-transfer reaction is exothermic with indene as a substrate (eqn (3)), but endothermic with other substrates. Thus, the enthalpy change for the charge-transfer between [Fe^IV^(O)(TPFPP^+^˙)]^+^ and indene should be equal to the difference between the ionization energy of the substrate and the electron affinity (EA) of [Fe^IV^(O)(TPFPP^+^˙)]^+^. Since, no charge-transfer reaction occurs with substrates with an ionization energy above 8.2 eV (Table 1), this implies that the EA of gaseous [Fe^IV^(O)(TPFPP^+^˙)]^+^ ions must be lower than 8.2 eV. Previous FT-ICR MS studies predicted a lower limit of 7.5 eV for the EA of [Fe^IV^(O)(TPFPP^+^˙)]^+^,^16^ which is in good general agreement with the data in Table 1. DFT calculations on [Fe^IV^(O)(Por^+^˙)X] with different axial ligands X have yielded electron affinities of 3.06 eV for X = SH^−^, whereas values of 6.41 and 6.12 eV are found for systems with an imidazole or tyrosinate axial ligand, respectively.^26^ Clearly, removal of the axial ligand is expected to raise the EA of the iron(iv)–oxo porphyrin cation radical substantially with respect to axially ligated systems due to the loss of interaction between the a_2u_ molecular orbital with axial ligand orbitals, *vide infra*.

3 [Fe^IV^(O)(TPFPP^+^˙)]^+^ + indene → [Fe^IV^(O)(TPFPP)] + indene^+^˙

4 Δ*H*_Eq3_ = IE_indene_ − EA_[Fe^IV^(O)(TPFPP^+^)]_ < 0 kcal mol^−1^

Interestingly, although cytochrome P450 isozymes react with typical olefins, such as propene, cyclohexene and *Z*- and *E*-butene, to give a mixture of epoxide and enol products, actually the precise product distributions depend on the specific P450 isozyme.^27^ Clearly, the substrate binding pocket and substrate orientation within the enzyme play a key role in determining the regioselectivity of the enzymatic reaction. In fact, enzymes manage to control the regioselectivity of substrate activation probably by binding the substrate under a specific orientation, which raises the epoxidation barriers and/or lowers the hydrogen atom abstraction barriers. Unfortunately, the FT-ICR MS results are unable to unequivocally distinguish hydroxylation products from epoxidation products as both have the same mass and are released as neutral molecules. As such, the experiments do not provide direct evidence supporting olefin epoxidation over a C–H activation channel in the reactions of [Fe^IV^(O)(TPFPP^+^˙)]^+^ with the selected olefins. However, we are able to show indirect evidence, *vide infra*. Computational studies on the regioselectivity of propene epoxidation *versus* hydroxylation by [Fe^IV^(O)(Por^+^˙)(SH)] gave lower epoxidation barriers in the gas-phase reaction,^28^ but solvent and environmental effects reversed the ordering. The computational studies from ref. 28, therefore, confirm the experimental trends in Table 1. This finding underlines the benchmark role played by a mechanistic study in the gas phase.

#### Theoretically derived reaction paths, energetics and structures

The experimental studies reported above present a comprehensive test set of model reactions of iron(iv)–oxo porphyrin cation radical complexes with olefins for the first time and enable extensive benchmarking and calibration of computational methods and procedures against gas-phase (Ideal Gas conditions) rate constants. We decided to take the opportunity and calibrate previously used methods and procedures for DFT studies on these chemical systems and compare to the results of the FT-ICR rates from Table 1. In addition, the computational studies were performed to further understand the substrate activation patterns by [Fe^IV^(O)(TPFPP^+^˙)]^+^ with olefins, and rationalize the obtained trends. Before we discuss details of the reaction mechanism and possible reactivity trends, let us start with a detailed analysis of the reactant species, namely [Fe^IV^(O)(Por^+^˙)]^+^, ^4,2^A, and [Fe^IV^(O)(TPFPP^+^˙)]^+^, ^4,2^B.

**Fig. 3:**
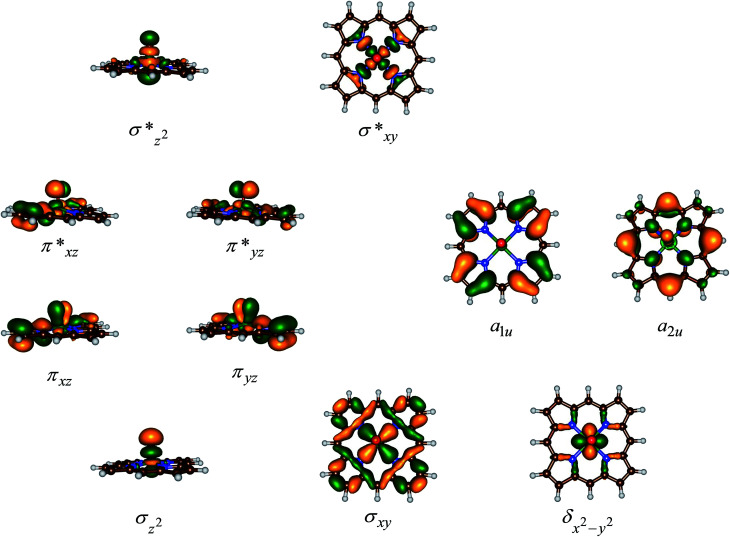
Molecular valence orbitals of ^4^A.

Fig. 3 displays the high-lying occupied and low-lying virtual orbitals of ^4,2^A; the orbitals for ^4,2^B look very similar. These orbitals are dominated by the interactions of the metal 3d orbitals with its ligands and several π-type porphyrin orbitals. Lowest in energy are a pair of σ-type orbitals (σ_*z*2_ and σ_*xy*_): σ_*z*2_ represents the σ-interactions of the 3d_*z*2_ orbital on iron with the 2p_*z*_ orbital on oxygen, whereas the σ_*xy*_ gives the interactions of the 3d_*xy*_ orbital on iron with 2p_*x*,*y*_ orbitals on the four nitrogen atoms of the porphyrin ligand. The antibonding combinations of these two orbitals (σ^*^_z2_ and σ^*^_xy_) are high in energy and virtual. Also doubly occupied is the δ_*x*2−*y*2_ orbital, which is a lone-pair orbital located in the plane of the porphyrin ring. Finally, the interaction of the metal 3d_*xz*_/3d_*yz*_ with the 2p_*x*_/2p_*y*_ on the oxygen atom leads to a pair of π_*xz*_/π_*yz*_ and a pair of π^*^_xz_/π^*^_yz_ set of orbitals. The σ_*z*2_, σ_*xy*_, π_*xz*_ and π_*yz*_ bonding orbitals are doubly occupied and low-lying in all calculations reported here. In addition to the metal-type orbitals there are also two porphyrin-type π-orbitals that in *D*_4h_ symmetry have the labels a_1u_ and a_2u_. With a thiolate as axial ligand the a_2u_ orbital strongly mixes with a 3p_*z*_ orbital on sulphur and hence is destabilized in energy,^29^ which strongly affects the electron affinity of the oxidant and consequently is responsible for its push-effect.^30^

The set of orbitals displayed in Fig. 3 is occupied with 15 electrons and as several of these orbitals are close in energy there are a number of possibilities to distribute the electrons over the orbitals. In addition, states can also be found in various spin states ranging from doublet to quartet and sextet, where we identify the spin state with a superscript in front of the electronic state label. Thus, the electronic state labelled as ^4^A_2u_ has an overall quartet spin state and singly occupied a_2u_ molecular orbital with overall electronic configuration: σ_*z*2_^2^ σ_*xy*_^2^ π_*xz*_^2^ π_*yz*_^2^ δ_*x*2−*y*2_^2^ π^*^_xz_^1^ π^*^_yz_^1^ a_1u_^2^ a_2u_^1^ or in short [core] δ_*x*2−*y*2_^2^ π^*^_xz_^1^ π^*^_yz_^1^ a_1u_^2^ a_2u_^1^. Similarly, we calculated the doublet spin state (^2^A_2u_ state), where the unpaired electron in the a_2u_ orbital is antiferromagnetically coupled to the unpaired electrons in the two π* orbitals: ^2^A_2u_ = [core] δ_*x*2−*y*2_^2^ π^*^_xz_^↑^ π^*^_yz_^↑^ a_1u_^2^ a_2u_^↓^.

Previous studies with either imidazole, acetonitrile or thiolate as axial ligand,^29,31^ showed the ^4,2^A_2u_ states to be close in energy and well below alternative states. However, this was due to considerable mixing of the a_2u_ orbital with the axial ligand orbitals, which obviously is not possible in our chemical system that lacks an axial ligand. However, in an isolated porphyrin macrocycle, the a_1u_ and a_2u_ orbitals are degenerate;^32^ therefore, we decided to investigate a range of possible electronic states for the pentacoordinated iron(iv)–oxo porphyrin cation radical system, [Fe^IV^(O)(Por^+^˙)]^+^. Firstly, we tested the stability of the ^4,2^A_2u_ states and the alternative ^4,2^A_1u_ states with [core] δ_*x*2−*y*2_^2^ π^*^_xz_^1^ π^*^_yz_^1^ a_1u_^1^ a_2u_^2^ orbital occupation. In addition, we attempted to generate models with the iron in oxidation state iron(v), *i.e.*^2^Π_*xz*_ state with occupation [core] δ_*x*2−*y*2_^2^ π^*^_xz_^1^ a_1u_^2^ a_2u_^2^, or the iron in oxidation state iron(iii), *i.e.* the ^4^A state with orbital occupation [core] δ_*x*2−*y*2_^2^ π^*^_xz_^2^ π^*^_yz_^1^ a_1u_^1^ a_2u_^1^ and the ^6^Σ_*xy*,III_ state with [core] δ_*x*2−*y*2_^2^ π^*^_xz_^1^ π^*^_yz_^1^ σ^*^_xy_^1^ a_1u_^1^ a_2u_^1^ occupation. However, all our attempts to calculate iron(iii) or iron(v) states failed and converged back to lower lying solutions with four electrons on the metal in a formal iron(iv) oxidation state, hence the ^2^Π_*xz*_, ^4^A and ^6^Σ_*xy*,III_ states are high in energy and inaccessible to our chemical system.

Table 2 summarizes relative energies of optimized geometries of the various electronic spin states as calculated with different DFT methods for ^2,4,6^A. As follows from Table 2 all calculations of A give a ^2^A_1u_ ground state that is nearly degenerate with the corresponding quartet spin state. In general, calculations done at UB3LYP/BS2 and UB3LYP-D3/BS2 give almost identical spin state orderings and relative energies, which shows that dispersion is not a critical component for these chemical structures. Nevertheless, the ^4,2^A_2u_ and ^4,2^A_1u_ states are close in energy and all four states could have a finite lifetime.

**Table 2:** Relative energies of several low-lying electronic states of [Fe(O)(Por^+^˙)]^+^ (A)^a^

State	Configuration	A^b^	A^c^	A^d^
		Δ*E* + ZPE	Δ*E* + ZPE	Δ*E* + ZPE
^2^A_1u_	δ^2^ π^*^_xz_^↑^ π^*^_yz_^↑^ a_1u_^↓^	0.00	0.00	0.00
^4^A_1u_	δ^2^ π^*^_xz_^↑^ π^*^_yz_^↑^ a_1u_^↑^	0.71	0.19	0.21
^2^A_2u_	δ^2^ π^*^_xz_^↑^ π^*^_yz_^↑^ a_2u_^↓^	1.65	3.75	3.80
^4^A_2u_	δ^2^ π^*^_xz_^↑^ π^*^_yz_^↑^ a_2u_^↑^	1.25	3.42	3.47
^6^A_2u_	δ^↑^ π^*^_xz_^↑^ π^*^_yz_^↑^ σ^*^_xy_^↑^ a_2u_^↑^	9.25	18.68	19.38
^4^Δ_*xy*_	δ^↑^ π^*^_xz_^↑^ π^*^_yz_^↑^ σ^*^_xy_^↑^ a_1u_^↓^	9.69	ND	ND
^4^Δ_*zz*_	δ^↑^ π^*^_xz_^↑^ π^*^_yz_^↑^ σ^*^_z2_^↑^ a_2u_^↓^	19.53	ND	ND

a Relative energies in kcal mol^−1^ with respect to the ^2^A_1u_ state, ND stands for not determined.

b Energies obtained at UB3LYP/BS2//UB3LYP/BS1 level of theory.

c Energies and geometries calculated at UB3LYP/BS2 level of theory.

d Energies and geometries calculated at UB3LYP-D3/BS2 level of theory.

**Fig. 4:**
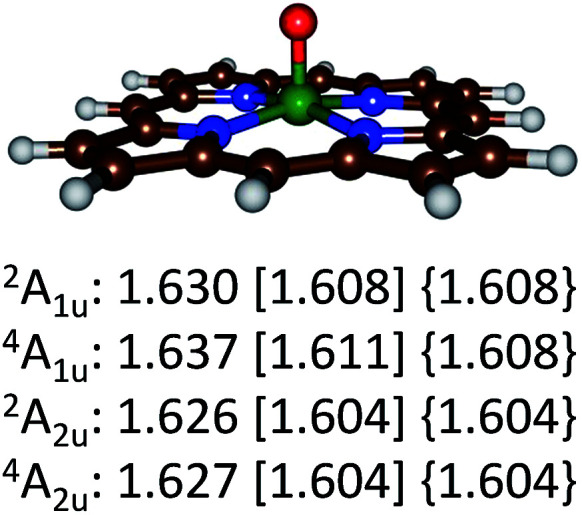
Optimized geometries of the ^4,2^A_2u_ and ^4,2^A_1u_ states of ^4,2^A as calculated with UB3LYP/BS1 [UB3LYP/BS2] {UB3LYP-D3/BS2} with Fe–O bond lengths in angstroms.

Optimized geometries of the ^4,2^A_2u_ and ^4,2^A_1u_ states are given in Fig. 4. Geometrically, no dramatic changes in bond lengths are obtained between the three optimization techniques. A small basis set gives slightly longer Fe–O distances than those found with a triple-ζ basis set. The effect of dispersion is negligible on the optimized geometries: UB3LYP/BS2 and UB3LYP-D3/BS2 give virtually the same chemical structures. Addition of *meso*-substituents to the porphyrin ring such as pentafluorophenyl groups is not expected to dramatically change key bond lengths in the optimized geometries and relative energies of individual spin states.^33^ Thus, recent work of the Goldberg group showed that *meso*-substituted manganese–oxo porphyrinoid complexes retained the spin state ordering and converged to a closed-shell singlet manganese(v)–oxo state in all cases.^33*a*^

The calculations on the low-lying ^4,2^A_2u_ and ^4,2^A_1u_ states reported in Table 2 and Fig. 4 show that geometrically there are very little differences between these states, but the spin state ordering and relative energies are sensitive to the method and basis set. Recent complete active site (CASSCF) and restricted active site (RASSCF) calculations of Pierloot and co-workers^34^ calculated the ^4,2^A_2u_ and ^4,2^A_1u_ states of A within 1 kcal mol^−1^ of each other with a small preference for the A_1u_ states. However, they also located two low-lying iron(v) states, which we were unable to characterize and for which no experimental evidence exist. Unfortunately, our chemical systems (in particular structure B) are too large to attempt calculations using the CASSCF and RASSCF methods; therefore, we decided to continue with UB3LYP instead.

Subsequently, we investigated the substrate epoxidation by [Fe^IV^(O)(Por^+^˙)]^+^, *i.e.*^4^A. We find the lowest lying barriers to proceed from the ^4^A_2u_ state and will focus on those in the following. Previous studies on the epoxidation of olefins by [Fe^IV^(O)(Por^+^˙)(L)] with L = NCCH_3_ or Cl^−^ showed that the same trends in reactivity are observed when the Por ligand is replaced by TPFPP,^35^ hence the smaller model was used in this study. We investigated substrate epoxidation with a range of olefins: ethene (1), propene (2), 1-butene (3), *E*-2-butene (4), cyclohexene (5), 1,3-cyclohexadiene (6), styrene (7), *trans*-β-methylstyrene (8), *Z*-2-butene (9) and 2-pinene (10). For all substrates we calculated the full potential energy profile from reactants to epoxide products, see ESI,† but for space restrictions we will focus on the rate determining C–O bond formation transition states (TS_CO_) only. All reactions are concerted with a single C–O activation barrier leading to epoxide product complexes P_E_. This is unusual as previous calculations on substrate epoxidation by Cpd I models gave a stepwise mechanism *via* a radical intermediate that *via* a ring-closure barrier was separated from epoxide product complexes.^36^ The orientation of the substrate and the strong displacement of the metal from the porphyrin plane are the likely reason for the fact that radical intermediates are saddlepoints here. Thus, the ring-closure barrier on the quartet spin state surface involves an electron transfer from substrate into σ^*^_z2_. The latter orbital in iron–porphyrin complexes with axial ligand, *e.g.* thiolate, contains a strong contribution from axial ligand orbitals (3p_*z*_) and therefore is high in energy. Since our particular system lacks an axial ligand, the σ^*^_z2_ orbital is considerably lower in energy and as a consequence the lifetime of the radical intermediate is reduced and the reaction to form products is now concerted.

**Fig. 5:**
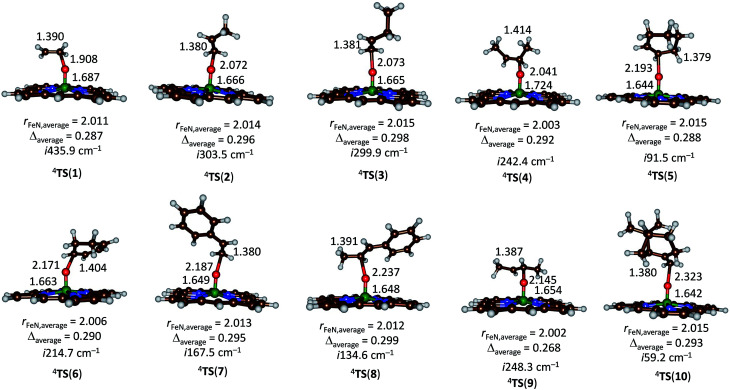
UB3LYP/BS1 optimized geometries of epoxidation transition states with bond lengths in angstroms.

Fig. 5 gives the optimized geometries of the C–O activation transition states (TS_CO_) for all substrates. Generally, the transition states occur early with a long C–O distance and relatively short Fe–O distance that has not dramatically changed from what it was in the iron(iv)–oxo porphyrin cation radical state. As expected the metal is considerably displaced from the plane through the four nitrogen atoms of the porphyrin ring by as much as 0.268–0.299 Å. These transition states bear resemblance to substrate epoxidation barriers calculated previously for P450 Cpd I reactions with olefins.^36^ Electronically, all transition states are accomplished by single electron transfer from the substrate into the a_2u_ orbital and the formation of an [Fe^IV^(OSub)(TPFPP)]^+^ transition state with orbital occupation [core] δ_*x*2−*y*2_^2^ π^*^_xz_^1^ π^*^_yz_^1^ a_1u_^2^ a_2u_^2^*ϕ*_Sub_^1^ with *ϕ*_Sub_ a radical on the substrate group.

## Discussion

The present work gives a detailed and extensive overview on the reactivity of iron(iv)–oxo porphyrin cation radical systems with a test-set of olefins. We determined rate constants and measured product ion distributions in the gas phase using FT-ICR MS. This comprehensive set of transition metal containing reactivities is unique and will enable computation to benchmark and calibrate its methods effectively. This is particularly important for transition metal complexes, such as iron(iv)-oxo species, where the reproducibility of the computational (DFT) methods sometimes varies strongly depending on the density functional method used, the basis set, environmental perturbations, dispersion effects *etc.*^37^ In this work, we supplemented the experimental studies with a series of preliminary DFT calculations for two reasons: (i) to validate and calibrate computational methods against experiment; (ii) to establish the physicochemical properties that influence the rate constant of the chemical reaction.

Let us first start with a comparison of the experimental and computational reaction rates. As FT-ICR MS experiments are being performed at very low pressures, these experimental conditions are close to Ideal Gas conditions with very few molecular collisions per second. In the kinetic study of ion-molecule reactions in the gas phase one needs to consider that thermal equilibration of the reacting system with the environment is in general not granted. On the contrary, when small species react at low pressures, the absence of thermalizing collisions leads to non-equilibrium energy distributions. In the absence of solvation, the double-well potential model first proposed by Brauman in 1977 to account for the kinetic behaviour of displacement reactions by anionic nucleophiles predicts that the energy of the intermediate and transition state must lie below the energy of the combined reactants.^38^ Because at low pressure the intermediates cannot be stabilized by unreactive collisions, determining the transition state energy is less straightforward than in solution. However, the kinetics results presently reported deal with a relatively large reactant ion that effectively establishes thermal equilibrium with the environment through coupling with the background radiation field allowed by the several low frequency infrared modes of the iron(iv)–oxo macrocyclic ligand complex. This condition is responsible, for example, for the consistency between the kinetics of NO ligand addition to iron(ii/iii) porphyrin complexes and the equilibrium data independently established through equilibrium measurements.^25^ Because of these considerations, the notion can be adopted that the presently investigated systems are in prevailing thermal equilibrium with the environment and reaction kinetics can be interpreted within the framework of transition state theory. Consequently, reaction rates represent bimolecular reactions and as such they should compare to computationally determined reaction rates well.

**Fig. 6:**
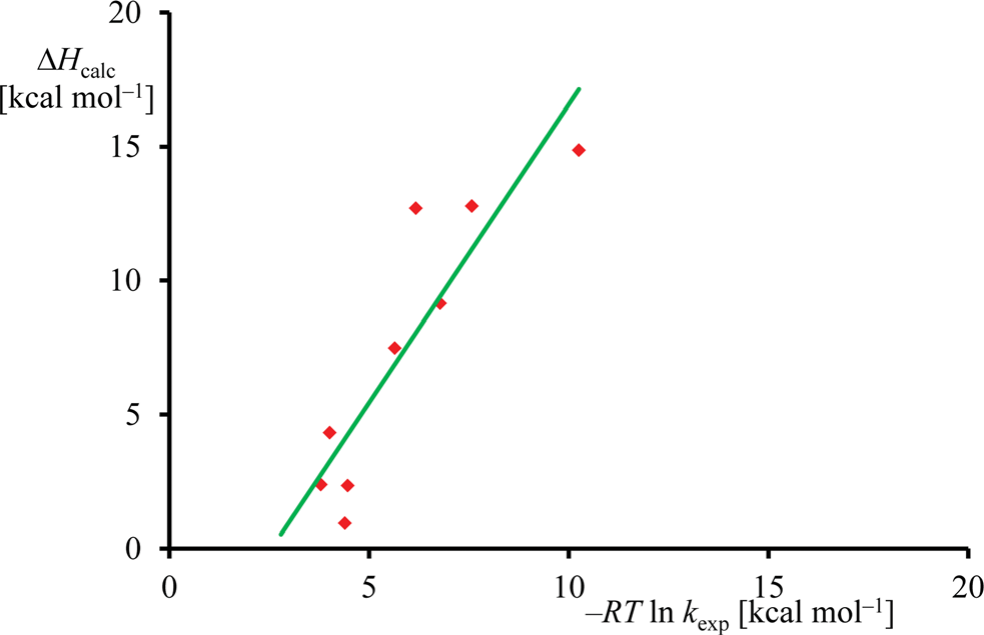
Correlation between experimental and computational barrier heights.

The experimental rate constants (Table 1) were converted into free energy units *via* −*RT* ln *k*_exp_, with *R* the gas constant and *T* the temperature, using transition state theory and plotted against the calculated enthalpy of activation for the same substrates, see Fig. 6. Although only a limited computational study is reported here, when we calculate the deviation between experiment and theory for each data point, we find an average difference between experiment and theory of 1.5 kcal mol^−1^ with a standard deviation of 3.4 kcal mol^−1^. As such, the DFT methods used here reproduce the trends obtained from experimental enthalpies of activation well and the linearity and reproducibility of the calculations is well within the typical error reported for DFT calculations using this method of about 5 kcal mol^−1^.^39^ There is, however, a large systematic error as well as a relatively large standard deviation that require further studies. Note that the experimental data in Fig. 6 refers to free energies of activation, whereas the computational results are enthalpy changes instead. The systematic error between experiment and theory contains entropic corrections to the energy.

**Fig. 7:**
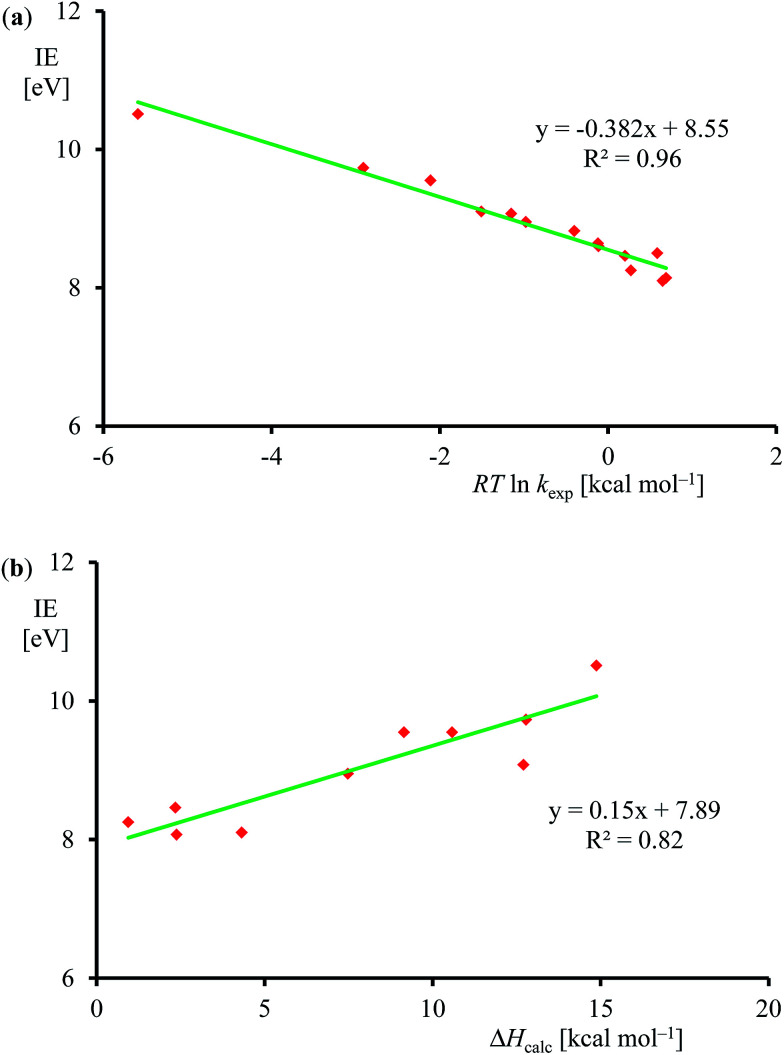
(a) Correlation between experimentally determined *RT* ln *k*_exp_ (for raw data, see Table 1) *versus* known ionization energies (IE). (b) Correlation between calculated epoxidation activation enthalpy (in kcal mol^−1^) and experimental ionization energy for the substrates in Fig. 5.

Subsequently, we investigated the origin of the rate constant, and in particular, the physical and chemical properties of the substrate and oxidant that determine the reaction mechanism and the enthalpy of activation of an epoxidation reaction. Previous studies on heteroatom oxidation and double bond epoxidation by P450 enzymes implicated a correlation between the natural logarithm of the rate constant with the ionization energy of the substrate.^12*d*,*e*,28,40^ To find out whether the data in Table 1 follow these trends as well, we plot *RT* ln *k*_exp_*versus* experimentally known ionization energies,^24^ in Fig. 7. The set of data shown in Table 1 and Fig. 7 gives a linear correlation between the natural logarithm of the rate constant and the ionization energy of the substrate with an *R*^2^ = 0.96. Fig. 7b displays the correlation between the DFT calculated enthalpy of activation of the reaction of [Fe^IV^(O)(Por^+^˙)]^+^ with olefins. In agreement with the experimental trends given in part (a) of Fig. 7 also the computational trends link the natural logarithm of the rate constant to the ionization energy of the substrate. Clearly, the key physicochemical property that drives the reaction mechanism and affects the rate constant of substrate epoxidation by iron(iv)–oxo porphyrin cation radical complexes is the ionization energy of the substrate.

**Fig. 8:**
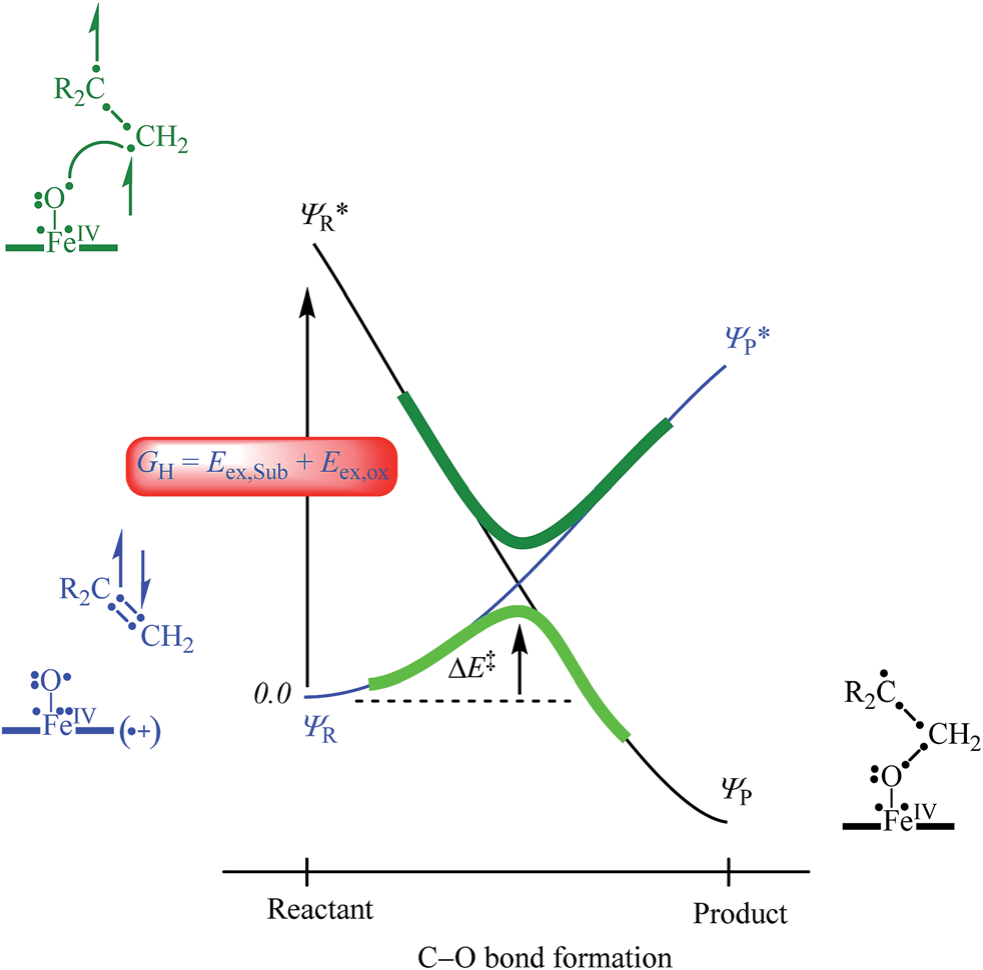
VB curve crossing diagram for the C–O bond formation step in olefin epoxidation (R_2_C=CH_2_) by [Fe^IV^(O)(TPFPP^+^˙)]^+^. Valence electrons are identified with a dot and lines (curved and straight) in the VB structures represent bonds.

In order to explain the experimental and computational trends in the reaction mechanisms, we devised a valence bond (VB) curve crossing diagram, which is schematically depicted in Fig. 8. This diagram starts bottom left with the reactant configuration of [Fe^IV^(O)(TPFPP^+^˙)]^+^ in electronic configuration π_*xz*_^2^ π_*yz*_^2^ δ_*x*2−*y*2_^2^ π^*^_xz_^1^ π^*^_yz_^1^ a_2u_^1^. The π and π* electrons along the FeO bond are identified with dots in the VB diagram and due to occupation of π_*xz*_^2^ π^*^_xz_^1^ there are three dots on the left-hand-side of the Fe–O bond. In addition, there are three electrons in the π_*yz*_ and π^*^_yz_ orbitals, which are identified with the other three dots on the right-hand-side of the Fe–O bond. Furthermore, the oxidant has a radical on the porphyrin ring for single occupation of the a_2u_ molecular orbital. The substrate double bond is also highlighted with four electrons spread out over the interaction. Upon approach of the substrate on the iron(iv)–oxo species a radical intermediate is formed that has a single bond between the oxygen and carbon atoms and a doubly occupied a_2u_ orbital.

In VB theory the electronic configuration in the reactant complex (*Ψ*_R_) connects to an excited state in the product geometry (Ψ^*^_P_) as shown with the blue line in Fig. 8. At the same time the product electronic configuration (*Ψ*_P_) connects to an excited state in the reactant geometry (Ψ^*^_R_), black line in Fig. 8. These two VB curves cross and lead to an avoided crossing and a transition state for the C–O bond formation with barrier Δ*E*^‡^. The barrier height is linearly proportional to the curve crossing energy, which in its own right is a fraction of the excitation energy (*G*_H_) from the reactant wave function to the product wave function in the geometry of the reactants, *i.e.* for *Ψ*_R_ → Ψ^*^_R_. The difference in VB structures for *Ψ*_R_ and Ψ^*^_R_ thereby should give a reflection of the key electron transfer/migrations upon product formation. Moreover, based on the excitation energy, the factors that determine the barrier height can be predicted.

An analysis of the differences between the reactant and product wave functions in the reactant geometry reveals the following information: First of all, a comparison of the VB structures of *Ψ*_R_ and Ψ^*^_R_ shows that the electrons in the π-bond of the olefin are singlet paired in the ground state and triplet coupled in the excited state, hence the excitation energy *G*_H_ includes the π–π* electron excitation in the substrate, *E*_ex,Sub_. Generally, the first ionization potential of an olefin corresponds to the removal of an electron from a π-orbital, and, hence, is proportional to the π–π* excitation energy. Indeed, our experimentally and computationally determined barrier heights correlate linearly with the ionization energy of the olefin, and therefore support the VB model.

One of the electrons originating from the π-bond of the olefin forms a bond with the π^*^_xz_ electron along the FeO bond, to create the C–O bonding pair of electrons. This means that the π_*xz*_/π^*^_xz_ pair of orbitals during the reaction splits back into individual atomic orbitals namely 3d_*xz*_(Fe) and 2p_*x*_(O). The 2p_*x*_(O) electron pairs with the electron from the substrate, while one of the electrons of the 3d_*xz*_(Fe) orbital is transferred into the a_2u_ orbital through internal excitation/rehybridization of the oxidant, *E*_ex,ox_. The promotion gap, *G*_H_, therefore, will be proportional to the π–π* excitation in the substrate and the 3d_*xz*_ to a_2u_ electron transfer in the oxidant: *G*_H_ = *E*_ex,Sub_ + *E*_ex,ox_. Obviously, since the ionization energy represents the energy to remove an electron from a π-type orbital of an olefin, this will imply a linear correlation between the first excited state and the ionization energy of the substrate.^28^ The VB diagram, therefore, confirms a linear correlation between the ionization energy of the substrate and the C–O bond formation enthalpy of activation as shown above.

**Fig. 9:**
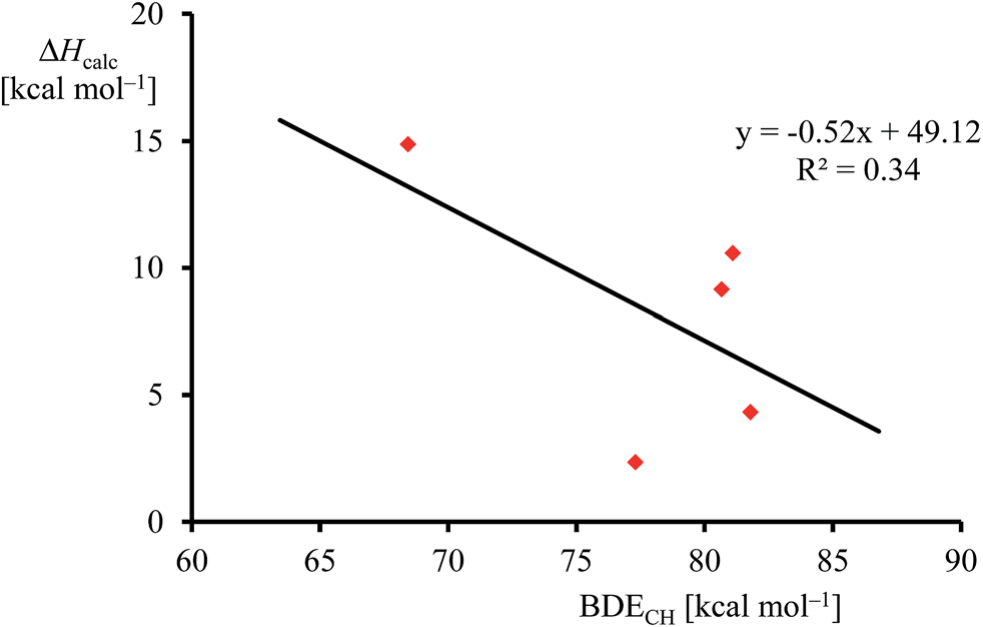
Correlation between calculated epoxidation activation enthalpy (in kcal mol^−1^) and BDE_CH_ for the substrates.

Although, the oxygen atom transfer reaction between [Fe^IV^(O)(TPFPP^+^˙)]^+^ and an olefin could lead to either epoxide or hydroxylated products, unfortunately the FT-ICR MS experiments cannot distinguish the two. Thus, several substrates in Scheme 2 and Table 1 contain aliphatic groups that in a reaction with an iron(iv)–oxo group can be converted into an alcohol. A correlation between the rate constant of oxygen atom transfer and the ionization energy, Fig. 7a, of the olefin provides indirect experimental evidence that all reactions lead to epoxidation products. In fact, hydrogen atom abstraction reactions should not correlate with the ionization potential of the substrate, but were shown to be proportional to the strength of the C–H bond of the substrate that is formed.^12*e*,40^ To test that the rate constants do not correlate with the bond dissociation energy (BDE_CH_) of the C–H bond of the substrate that is broken, we plot in Fig. 9 calculated BDE_CH_ and barrier heights of selected olefins, namely propene, *Z*-2-butene, *E*-2-butene, cyclohexene and 1,3-cyclohexadiene. As can be seen from Fig. 9, no correlation between BDE_CH_ and barrier height exists, and, therefore, hydrogen atom abstraction is not the rate determining step in the reaction mechanism. Further evidence that hydrogen atom abstraction reactions can be ruled out here comes from kinetic isotope effect (KIE) studies. We measured the rate constant of oxygen atom transfer with cyclohexene and cyclohexene-*d*_10_ and determined a KIE = *k*_H_/*k*_D_ ∼1 (Table 1). Consequently, the oxygen atom transfer is unlikely to proceed with an initial hydrogen atom abstraction and double bond epoxidation will be the dominant pathway.

The experimental trends, therefore, provide the first indirect experimental evidence that in the gas-phase the regioselectivity of double bond epoxidation *versus* aliphatic hydroxylation will be in favour of the epoxidation pathway. This implies that in enzymatic systems, such as the cytochromes P450, the shape and size of the substrate binding pocket will influence the regioselectivity of hydroxylation over epoxidation and can change the natural preference away from epoxidation.

Finally, the calculations presented in this work obviously refer to gas-phase results and hence correlate well with gas-phase mass spectrometric data. In order to further establish that the work can be extrapolated to solution phase, we did a series of single point calculations using the polarized continuum model with a dielectric constant of *ε* = 4.7 to mimic a solution. The obtained correlation between solvent corrected free energies of activation of epoxidation reactions by [Fe^IV^(O)(Por^+^˙)]^+^ is plotted against the solvent corrected ionization energy of all substrates in Fig. S1, ESI.† Even in solvent, the linear trend in the correlation between free energy of activation and ionization energy is retained, therefore, we expect to be able to extrapolate our results to the solution phase as well.

## Conclusions

In this work we report a comprehensive combined mass spectrometric and computational study on substrate epoxidation by iron(iv)–oxo porphyrin cation radical complexes in the gas phase. We present a novel method to synthesize [Fe^IV^(O)(TPFPP^+^˙)]^+^ in the gas phase at low pressure. Furthermore, we report a large set of experimentally derived rate constants and product distributions. All olefins undergo oxygen atom transfer, whereas compounds with low ionization energy also give a certain degree of hydride transfer and charge transfer reactions. Our experimentally determined reaction rates correlate linearly with the ionization potential of the substrate and show that the electron transfer from substrate to oxidant is rate determining. A thorough computational survey has confirmed the suggested mechanism and provides a rationale for the observed trend in the rate constants. Moreover, the work highlights a regioselective epoxidation over hydroxylation in the gas phase.

## Supplementary Material

SC-006-C4SC02717E-s001
