# Supplementary material for: A comprehensive test set of epoxidation rate constants for iron(iv)–oxo porphyrin cation radical complexes
Source: Chem Sci. 2014 Dec 8;6(2):1516–29. doi: 10.1039/c4sc02717e (PMC5811088; doi:10.1039/c4sc02717e)
Supplement: SC-006-C4SC02717E-s001 [file SC-006-C4SC02717E-s001.pdf]

Electronic Supplementary Material (ESI) for Chemical Science.
This journal is © The Royal Society of Chemistry 2014

Electronic Supporting Information

A comprehensive test set of epoxidation rate constants by iron(IV)-oxo porphyrin complexes

Mala A. Sainna,[a] Suresh Kumar,[b] Devesh Kumar,*[b] Simonetta Fornarini,*[c] Maria Elisa Crestoni*[c] and Sam P. de Visser*[a]

[a] Manchester Institute of Biotechnology and School of Chemical Engineering and Analytical Science, The University of Manchester, 131 Princess Street, Manchester M1 7DN, United Kingdom. E-mail: sam.devisser@manchester.ac.uk

[b] Department of Applied Physics, School for Physical Sciences, Babasaheb Bhimrao Ambedkar University, Vidya Vihar, Rai Bareilly Road, Lucknow 226 025, India. E-mail: dkclcre@yahoo.com

[c] Dipartimento di Chimica e Tecnologie del Farmaco, Università di Roma "La Sapienza", P.le A. Moro 5, 00185, Roma, Italy, E-mail: mariaelisa.crestoni@uniroma1.it / simonetta.fornarini@uniroma1.it

Table S1: Absolute energies, zero-point energies and free energies (in au) of optimized geometries of different electronic states of $[Fe^{IV}(O)(Por^{+\bullet})]^+$ at UB3LYP/BS1 in Jaguar.

		E, au	ZPE, au	G, au	E, au
		[BS1]	[BS1]	[BS1]	[BS2]
$^4\Delta_{xy}$	$\delta^{\uparrow}\ \pi^*_{xz}{}^{\uparrow}\ \pi^*_{yz}{}^{\uparrow}\ \sigma^*_{xy}{}^{\uparrow}\ a_{1u}{}^{\downarrow}$	-1186.51601	0.27703	-1186.28601	-1187.04719
$^2A_{1u}$	$\delta^2\ \pi^*_{xz}{}^{\uparrow}\ \pi^*_{yz}{}^{\uparrow}\ a_{1u}{}^{\downarrow}$	-1186.53700	0.27858	-1186.30589	-1187.06418
$^4A_{1u}$	$\delta^2\ \pi^*_{xz}{}^{\uparrow}\ \pi^*_{yz}{}^{\uparrow}\ a_{1u}{}^{\uparrow}$	-1186.53657	0.27834	-1186.30479	-1187.06282
$^2A_{2u}$	$\delta^2\ \pi^*_{xz}{}^{\uparrow}\ \pi^*_{yz}{}^{\uparrow}\ a_{2u}{}^{\downarrow}$	-1186.53360	0.27862	-1186.30109	-1187.06160
$^4A_{2u}$	$\delta^2\ \pi^*_{xz}{}^{\uparrow}\ \pi^*_{yz}{}^{\uparrow}\ a_{2u}{}^{\uparrow}$	-1186.53428	0.27864	-1186.30238	-1187.06224
$^6A_{2u}$	$\delta^{\uparrow}\ \pi^*_{xz}{}^{\uparrow}\ \pi^*_{yz}{}^{\uparrow}\ \sigma^*_{xy}{}^{\uparrow}\ a_{2u}{}^{\uparrow}$	-1186.51494	0.27647	-1186.28603	-1187.04733
$^4\Delta_{zz}$	$\delta^{\uparrow}\ \pi^*_{xz}{}^{\uparrow}\ \pi^*_{yz}{}^{\uparrow}\ \sigma^*_{zz}{}^{\uparrow}\ a_{2u}{}^{\downarrow}$	-1186.50682	0.28014	-1186.27230	-1187.03461

Table S2: Relative energies and free energies (in kcal mol^{-1}) of optimized geometries of different electronic states of $[Fe^{IV}(O)(Por^{+\bullet})]^+$ at UB3LYP/BS1 in Jaguar.

	ΔE	ΔE+ZPE	ΔE	ΔE+ZPE	ΔG	ΔG
	[BS1]	[BS1]	[BS2]	[BS2]	[BS1]	[BS2]
$^4\Delta_{xy}$	13.17	12.20	10.66	9.69	12.47	9.96
$^2A_{1u}$	0.00	0.00	0.00	0.00	0.00	0.00
$^4A_{1u}$	0.27	0.12	0.86	0.71	0.69	1.28
$^2A_{2u}$	2.14	2.16	1.62	1.65	3.02	2.50
$^4A_{2u}$	1.70	1.74	1.22	1.25	2.21	1.72
$^6A_{2u}$	13.84	12.52	10.58	9.25	12.47	9.20
$^4\Delta_{zz}$	18.94	19.92	18.55	19.53	21.08	20.69

Table S3: Absolute energies (in au) and relative energies (in kcal mol^{-1}) of optimized geometries of different electronic states of $[Fe^{IV}(O)(Por^{+\bullet})]^+$ at UB3LYP/BS2 in Jaguar.

		E, au	ΔE
		[BS2]	[BS2]
$^2A_{2u}$	$\delta^2\ \pi^*_{xz}{}^{\uparrow}\ \pi^*_{yz}{}^{\uparrow}\ a_{2u}{}^{\downarrow}$	-1187.06414	3.72
$^4A_{2u}$	$\delta^2\ \pi^*_{xz}{}^{\uparrow}\ \pi^*_{yz}{}^{\uparrow}\ a_{2u}{}^{\uparrow}$	-1187.06468	3.38
$^6A_{2u}$	$\delta^{\uparrow}\ \pi^*_{xz}{}^{\uparrow}\ \pi^*_{yz}{}^{\uparrow}\ \sigma^*_{xy}{}^{\uparrow}\ a_{2u}{}^{\uparrow}$	-1187.03820	20.00
$^2A_{1u}$	$\delta^2\ \pi^*_{xz}{}^{\uparrow}\ \pi^*_{yz}{}^{\uparrow}\ a_{1u}{}^{\downarrow}$	-1187.07007	0.00
$^4A_{1u}$	$\delta^2\ \pi^*_{xz}{}^{\uparrow}\ \pi^*_{yz}{}^{\uparrow}\ a_{1u}{}^{\uparrow}$	-1187.06952	0.34

Table S4: Absolute energies (in au) and relative energies (in kcal mol^{-1}) of optimized geometries of different electronic states of $[Fe^{IV}(O)(Por^{+\bullet})]^+$ at UB3LYP-D3/BS2 in Jaguar.

		E, au	ΔE
		[BS2]	[BS2]
$^2A_{2u}$	$\delta^2\ \pi^*_{xz}{}^{\uparrow}\ \pi^*_{yz}{}^{\uparrow}\ a_{2u}{}^{\downarrow}$	-1187.110789	3.78
$^4A_{2u}$	$\delta^2\ \pi^*_{xz}{}^{\uparrow}\ \pi^*_{yz}{}^{\uparrow}\ a_{2u}{}^{\uparrow}$	-1187.111331	3.44
$^6A_{2u}$	$\delta^{\uparrow}\ \pi^*_{xz}{}^{\uparrow}\ \pi^*_{yz}{}^{\uparrow}\ \sigma^*_{xy}{}^{\uparrow}\ a_{2u}{}^{\uparrow}$	-1187.083809	20.71
$^2A_{1u}$	$\delta^2\ \pi^*_{xz}{}^{\uparrow}\ \pi^*_{yz}{}^{\uparrow}\ a_{1u}{}^{\downarrow}$	-1187.116807	0.00
$^4A_{1u}$	$\delta^2\ \pi^*_{xz}{}^{\uparrow}\ \pi^*_{yz}{}^{\uparrow}\ a_{1u}{}^{\uparrow}$	-1187.116237	0.36

Table S5: Group spin densities and charges of optimized geometries of different electronic states of $[Fe^{IV}(O)(Por^{+\bullet})]^+$ at UB3LYP/BS1 in Jaguar.

		Spin Densities			Charges		
		Fe	O	Por	Fe	O	Por
$^4\Delta_{xy}$	$\delta^{\uparrow}\ \pi^*_{xz}{}^{\uparrow}\ \pi^*_{yz}{}^{\uparrow}\ \sigma^*_{xy}{}^{\uparrow}\ a_{1u}{}^{\downarrow}$	3.33	0.45	-0.78	0.98	-0.34	0.35
$^2A_{1u}$	$\delta^2\ \pi^*_{xz}{}^{\uparrow}\ \pi^*_{yz}{}^{\uparrow}\ a_{1u}{}^{\downarrow}$	1.12	0.90	-1.02	0.77	-0.28	0.51
$^4A_{1u}$	$\delta^2\ \pi^*_{xz}{}^{\uparrow}\ \pi^*_{yz}{}^{\uparrow}\ a_{1u}{}^{\uparrow}$	1.15	0.90	0.95	0.77	-0.28	0.52
$^2A_{2u}$	$\delta^2\ \pi^*_{xz}{}^{\uparrow}\ \pi^*_{yz}{}^{\uparrow}\ a_{2u}{}^{\downarrow}$	1.09	0.89	-0.99	0.77	-0.26	0.49
$^4A_{2u}$	$\delta^2\ \pi^*_{xz}{}^{\uparrow}\ \pi^*_{yz}{}^{\uparrow}\ a_{2u}{}^{\uparrow}$	1.13	0.91	0.95	0.77	-0.26	0.49
$^6A_{2u}$	$\delta^{\uparrow}\ \pi^*_{xz}{}^{\uparrow}\ \pi^*_{yz}{}^{\uparrow}\ \sigma^*_{xy}{}^{\uparrow}\ a_{2u}{}^{\uparrow}$	3.29	0.47	1.25	1.00	-0.31	0.31
$^4\Delta_{zz}$	$\delta^{\uparrow}\ \pi^*_{xz}{}^{\uparrow}\ \pi^*_{yz}{}^{\uparrow}\ \sigma^*_{zz}{}^{\uparrow}\ a_{2u}{}^{\downarrow}$	2.43	1.07	-0.50	0.93	-0.26	0.33

Table S6: Group spin densities and charges of different electronic states of $[Fe^{IV}(O)(Por^{+\bullet})]^+$ calculated at UB3LYP/BS2//UB3LYP/BS1 in Jaguar.

		Spin Densities			Charges		
		Fe	O	Por	Fe	O	Por
$^4\Delta_{xy}$	$\delta^{\uparrow}\ \pi^*_{xz}{}^{\uparrow}\ \pi^*_{yz}{}^{\uparrow}\ \sigma^*_{xy}{}^{\uparrow}\ a_{1u}{}^{\downarrow}$	3.50	0.61	-1.11	0.60	-0.19	0.60
$^2A_{1u}$	$\delta^2\ \pi^*_{xz}{}^{\uparrow}\ \pi^*_{yz}{}^{\uparrow}\ a_{1u}{}^{\downarrow}$	1.16	0.88	-1.04	0.40	-0.18	0.78
$^4A_{1u}$	$\delta^2\ \pi^*_{xz}{}^{\uparrow}\ \pi^*_{yz}{}^{\uparrow}\ a_{1u}{}^{\uparrow}$	1.13	0.92	0.95	0.40	-0.18	0.78
$^2A_{2u}$	$\delta^2\ \pi^*_{xz}{}^{\uparrow}\ \pi^*_{yz}{}^{\uparrow}\ a_{2u}{}^{\downarrow}$	1.18	0.85	-1.02	0.29	-0.12	0.84
$^4A_{2u}$	$\delta^2\ \pi^*_{xz}{}^{\uparrow}\ \pi^*_{yz}{}^{\uparrow}\ a_{2u}{}^{\uparrow}$	1.18	0.87	0.95	0.28	-0.13	0.84
$^6A_{2u}$	$\delta^{\uparrow}\ \pi^*_{xz}{}^{\uparrow}\ \pi^*_{yz}{}^{\uparrow}\ \sigma^*_{xy}{}^{\uparrow}\ a_{2u}{}^{\uparrow}$	3.41	0.41	1.18	0.51	-0.11	0.60
$^4\Delta_{zz}$	$\delta^{\uparrow}\ \pi^*_{xz}{}^{\uparrow}\ \pi^*_{yz}{}^{\uparrow}\ \sigma^*_{zz}{}^{\uparrow}\ a_{2u}{}^{\downarrow}$	2.40	0.94	-0.34	0.39	-0.08	0.69

Table S7: Group spin densities and charges of optimized geometries of different electronic states of $[Fe^{IV}(O)(Por^{+\bullet})]^+$ at UB3LYP/BS2 in Jaguar.

		Spin Densities			Charges		
		Fe	O	Por	Fe	O	Por
$^2A_{1u}$	$\delta^2\ \pi^*_{xz}{}^{\uparrow}\ \pi^*_{yz}{}^{\uparrow}\ a_{1u}{}^{\downarrow}$	1.20	0.84	-1.04	0.33	-0.14	0.81
$^4A_{1u}$	$\delta^2\ \pi^*_{xz}{}^{\uparrow}\ \pi^*_{yz}{}^{\uparrow}\ a_{1u}{}^{\uparrow}$	1.20	0.86	0.95	0.32	-0.14	0.82
$^2A_{2u}$	$\delta^2\ \pi^*_{xz}{}^{\uparrow}\ \pi^*_{yz}{}^{\uparrow}\ a_{2u}{}^{\downarrow}$	1.19	0.83	-1.02	0.29	-0.12	0.82
$^4A_{2u}$	$\delta^2\ \pi^*_{xz}{}^{\uparrow}\ \pi^*_{yz}{}^{\uparrow}\ a_{2u}{}^{\uparrow}$	1.19	0.85	0.96	0.29	-0.12	0.83
$^6A_{2u}$	$\delta^{\uparrow}\ \pi^*_{xz}{}^{\uparrow}\ \pi^*_{yz}{}^{\uparrow}\ \sigma^*_{xy}{}^{\uparrow}\ a_{2u}{}^{\uparrow}$	4.12	1.37	-0.49	0.50	-0.10	0.60

Table S8: Group spin densities and charges of optimized geometries of different electronic states of $[Fe^{IV}(O)(Por^{+\bullet})]^+$ at UB3LYP-D3/BS2 in Jaguar.

		Spin Densities			Charges		
		Fe	O	Por	Fe	O	Por
$^2A_{1u}$	$\delta^2\ \pi^*_{xz}{}^{\uparrow}\ \pi^*_{yz}{}^{\uparrow}\ a_{1u}{}^{\downarrow}$	1.20	0.84	-1.04	0.33	-0.14	0.81
$^4A_{1u}$	$\delta^2\ \pi^*_{xz}{}^{\uparrow}\ \pi^*_{yz}{}^{\uparrow}\ a_{1u}{}^{\uparrow}$	1.24	0.82	0.94	0.32	-0.15	0.82
$^2A_{2u}$	$\delta^2\ \pi^*_{xz}{}^{\uparrow}\ \pi^*_{yz}{}^{\uparrow}\ a_{2u}{}^{\downarrow}$	1.19	0.83	-1.02	0.29	-0.12	0.83
$^4A_{2u}$	$\delta^2\ \pi^*_{xz}{}^{\uparrow}\ \pi^*_{yz}{}^{\uparrow}\ a_{2u}{}^{\uparrow}$	1.19	0.85	0.96	0.29	-0.12	0.83
$^6A_{2u}$	$\delta^{\uparrow}\ \pi^*_{xz}{}^{\uparrow}\ \pi^*_{yz}{}^{\uparrow}\ \sigma^*_{xy}{}^{\uparrow}\ a_{2u}{}^{\uparrow}$	4.13	1.36	-0.49	0.51	-0.10	0.59

Table S9: Group spin densities of optimised Reactant complexes for the epoxidation reaction of $[Fe^{IV}(O)(Por^{+\bullet})]^+$ with olefins as calculated at UB3LYP/BS1 in Gaussian.

**Spin**	Fe	O	Por	Sub
**$^4Re_1$**	1.22	0.82	0.93	0.03
**$^4Re_2$**	1.18	0.87	0.90	0.05
**$^4Re_3$**	1.16	0.88	0.96	0.00
**$^4Re_4$**	1.20	0.84	0.79	0.17
**$^4Re_5$**	1.20	0.84	0.77	0.19
**$^4Re_6$**	1.20	0.82	0.66	0.32
**$^4Re_7$**	1.19	0.85	0.66	0.31
**$^4Re_8$**	1.27	0.78	0.46	0.51
**$^4Re_9$**	1.20	0.84	0.79	0.17
**$^4Re_{10}$**	1.22	0.82	0.69	0.26

Table S10: Group charges of optimised Reactant complexes for the epoxidation reaction of $[Fe^{IV}(O)(Por^{+\bullet})]^+$ with olefins as calculated at UB3LYP/BS1 in Gaussian.

Charge	Fe	O	Por	Sub
$^4Re_1$	0.9	-0.3	0.3	0.1
$^4Re_2$	1.0	-0.3	0.3	0.1
$^4Re_3$	1.1	-0.3	0.2	0.0
$^4Re_4$	1.0	-0.4	0.0	0.4
$^4Re_5$	0.5	0.0	0.4	0.1
$^4Re_6$	1.0	-0.4	0.1	0.3
$^4Re_7$	0.9	-0.3	0.1	0.3
$^4Re_8$	0.5	0.1	0.2	0.3
$^4Re_9$	0.5	-0.1	0.6	0.0
$^4Re_{10}$	0.5	-0.0	0.4	0.2

Table S11: Group spin densities of optimised Transition states complexes for the epoxidation reaction of $[Fe^{IV}(O)(Por^{+\bullet})]^{+}$ with olefins as calculated at UB3LYP/BS1 in Gaussian.

**Spin**	Fe	O	Por	Sub
$^{4}\boldsymbol{TS_{1}}$	1.2	0.7	0.6	0.5
$^{4}\boldsymbol{TS_{2}}$	1.4	0.6	0.6	0.4
$^{4}\boldsymbol{TS_{3}}$	1.4	0.6	0.5	0.4
$^{4}\boldsymbol{TS_{4}}$	1.5	0.6	0.4	0.5
$^{4}\boldsymbol{TS_{5}}$	1.3	0.8	0.6	0.4
$^{4}\boldsymbol{TS_{6}}$	1.2	0.8	0.4	0.5
$^{4}\boldsymbol{TS_{7}}$	1.4	0.7	0.4	0.6
$^{4}\boldsymbol{TS_{8}}$	1.4	0.7	0.3	0.7
$^{4}\boldsymbol{TS_{9}}$	1.3	0.7	0.4	0.5
$^{4}\boldsymbol{TS_{10}}$	1.3	0.7	0.5	0.5

Table S12: Group charges of optimised Transition states complexes for the epoxidation reaction of $[Fe^{IV}(O)(Por^{+\bullet})]^{+}$ with olefins as calculated at UB3LYP/BS1 in Gaussian.

Charge	Fe	O	Por	Sub
$^{4}\boldsymbol{TS_{1}}$	1.0	0.2	0.1	-0.3
$^{4}\boldsymbol{TS_{2}}$	1.1	-0.7	0.3	0.4
$^{4}\boldsymbol{TS_{3}}$	0.5	0.1	0.4	0.1
$^{4}\boldsymbol{TS_{4}}$	0.5	0.6	0.2	-0.2
$^{4}\boldsymbol{TS_{5}}$	0.5	0.4	0.2	-0.1
$^{4}\boldsymbol{TS_{6}}$	1.3	-1.2	0.2	0.7
$^{4}\boldsymbol{TS_{7}}$	0.4	0.5	0.1	0.0
$^{4}\boldsymbol{TS_{8}}$	1.0	-0.4	-0.2	0.7
$^{4}\boldsymbol{TS_{9}}$	0.5	0.1	0.4	0.1
$^{4}\boldsymbol{TS_{10}}$	0.5	0.4	0.1	0.0

Table S13: Group spin densities of optimised Product complexes for the epoxidation reaction of $[Fe^{IV}(O)(Por^{+\bullet})]^+$ with olefins as calculated at UB3LYP/BS1 in Gaussian.

Spin	Fe	O	Por	Sub
$^4P_1$	2.79	0.08	0.11	0.02
$^4P_2$	2.88	0.06	0.04	0.02
$^4P_3$	2.79	0.07	0.10	0.03
$^4P_4$	2.78	0.10	0.10	0.02
$^4P_5$	2.90	0.07	0.01	0.02
$^4P_6$	2.80	0.08	0.10	0.02
$^4P_7$	2.78	0.08	0.11	0.04
$^4P_8$	2.80	0.06	0.10	0.05
$^4P_9$	2.79	0.08	0.10	0.03
$^4P_{10}$	2.80	0.07	0.09	0.04

Table S14: Group spin densities of optimised Product complexes for the epoxidation reaction of $[Fe^{IV}(O)(Por^{+\bullet})]^+$ with olefins as calculated at UB3LYP/BS1 in Gaussian.

Charge	Fe	O	Por	Sub
$^4P_1$	0.9	-0.6	0.0	0.6
$^4P_2$	0.9	-0.6	0.0	0.7
$^4P_3$	1.0	-0.7	-0.1	0.8
$^4P_4$	1.0	-0.7	0.0	0.8
$^4P_5$	0.9	-0.7	0.0	0.7
$^4P_6$	1.0	-0.8	-0.1	0.8
$^4P_7$	1.0	-0.7	-0.1	0.8
$^4P_8$	1.1	-0.9	-0.1	0.9
$^4P_9$	1.0	-0.7	-0.1	0.8
$^4P_{10}$	1.1	-0.9	-0.1	0.9

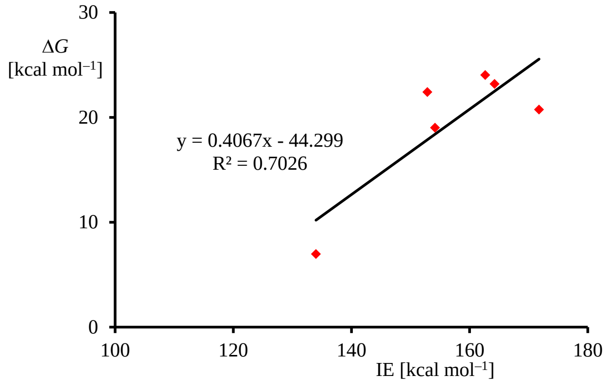

Figure S1: Correlation between solvent corrected free energies of activation and solvent corrected ionization energies for substrate epoxidation by $[Fe^{IV}(O)(Por^{+\bullet})]^{+}$.

Cartesian coordinates of optimized geometries:

UB3LYP/BS1 optimized structures of $[Fe^{IV}(O)(Por^{+\bullet})]^{+}$:

$^{2}A_{1u}$:

26	-0.103433000	0.135553000	0.033144000
7	-0.319522000	-0.021819000	2.019391000
7	1.884889000	0.088531000	0.277088000
7	-2.034934000	-0.349912000	-0.182362000
7	0.162366000	-0.179309000	-1.928269000
6	-1.511424000	-0.092741000	2.726576000
6	2.852098000	0.137593000	-0.722406000
6	0.669385000	0.151465000	2.982489000
6	2.583910000	0.223554000	1.468458000
6	-3.001320000	-0.405382000	0.817867000
6	1.350821000	-0.073193000	-2.636624000
6	-2.734062000	-0.486899000	-1.373767000
6	-0.830572000	-0.312985000	-2.893620000
6	-1.261448000	0.031473000	4.160145000
6	4.182141000	0.276627000	-0.134345000
6	0.076328000	0.182847000	4.317500000
6	4.017069000	0.330592000	1.209553000
6	-4.323893000	-0.612435000	0.233255000
6	1.096503000	-0.153213000	-4.072619000
6	-4.159864000	-0.659493000	-1.110982000
6	-0.241925000	-0.297583000	-4.230878000
1	-2.029463000	-0.001064000	4.918043000
1	5.098525000	0.324047000	-0.703149000
1	0.638299000	0.301758000	5.231700000
1	4.769087000	0.432804000	1.977140000
1	-5.235344000	-0.703982000	0.804515000
1	1.863145000	-0.104739000	-4.831051000
1	-4.908241000	-0.796383000	-1.876544000
1	-0.806062000	-0.391829000	-5.146620000
6	-2.768700000	-0.275373000	2.172383000
6	2.612209000	0.072296000	-2.080362000
6	-2.180659000	-0.458735000	-2.644416000
6	2.023075000	0.263765000	2.735494000
1	-3.618493000	-0.324502000	2.842321000
1	3.460480000	0.132872000	-2.751313000
1	-2.847107000	-0.563432000	-3.491977000
1	2.687666000	0.387439000	3.581980000
8	-0.274177000	1.754478000	-0.055472000

$^{4}A_{1u}$:

26	-0.104054000	0.144806000	0.032334000
7	-0.318981000	-0.005999000	2.019862000
7	1.881966000	0.095911000	0.277206000
7	-2.031477000	-0.349676000	-0.183732000
7	0.159387000	-0.155891000	-1.930506000
6	-1.516162000	-0.033125000	2.722380000
6	2.844853000	0.171426000	-0.721860000
6	0.670648000	0.130768000	2.984737000
6	2.586877000	0.176885000	1.473108000
6	-3.000049000	-0.370235000	0.813029000
6	1.343927000	-0.007447000	-2.639571000
6	-2.724799000	-0.540436000	-1.373261000
6	-0.826328000	-0.333594000	-2.893663000
6	-1.266017000	0.089613000	4.157503000
6	4.178935000	0.272818000	-0.134327000
6	0.075621000	0.184403000	4.318461000
6	4.020860000	0.270027000	1.211223000
6	-4.319646000	-0.607548000	0.232689000
6	1.089227000	-0.090433000	-4.076672000
6	-4.149807000	-0.718937000	-1.106733000
6	-0.240685000	-0.296199000	-4.231993000
1	-2.037501000	0.091226000	4.912673000
1	5.093001000	0.333775000	-0.705506000
1	0.639741000	0.281888000	5.233496000
1	4.777233000	0.329366000	1.978974000
1	-5.232621000	-0.675757000	0.804451000
1	1.851749000	-0.007065000	-4.836313000
1	-4.893920000	-0.897011000	-1.868034000
1	-0.802105000	-0.416788000	-5.146140000
6	-2.772040000	-0.198141000	2.166275000
6	2.600825000	0.149854000	-2.083348000
6	-2.171618000	-0.531127000	-2.641378000
6	2.030160000	0.199172000	2.739255000
1	-3.625654000	-0.217529000	2.832735000
1	3.447368000	0.237073000	-2.753559000
1	-2.833355000	-0.675725000	-3.486707000
1	2.697962000	0.285070000	3.587834000
8	-0.288421000	1.769544000	-0.039665000

$^{2}A_{2u}$:

26	-0.103462000	0.131529000	0.033848000
7	-0.321699000	-0.008460000	2.034736000
7	1.898445000	0.085660000	0.278586000
7	-2.048185000	-0.351877000	-0.183348000
7	0.162057000	-0.166253000	-1.945090000
6	-1.515351000	-0.073470000	2.742523000
6	2.864230000	0.135557000	-0.715011000
6	0.661108000	0.159537000	2.996730000
6	2.600027000	0.212644000	1.471591000
6	-3.012938000	-0.409668000	0.811080000
6	1.351339000	-0.058075000	-2.654505000
6	-2.747261000	-0.499418000	-1.375580000
6	-0.823888000	-0.303734000	-2.908810000
6	-1.270728000	0.046487000	4.157527000
6	4.177849000	0.270948000	-0.141017000
6	0.081850000	0.190038000	4.315423000
6	4.013920000	0.318641000	1.218000000
6	-4.318482000	-0.617826000	0.240583000
6	1.102904000	-0.137538000	-4.071847000
6	-4.153553000	-0.673654000	-1.118041000
6	-0.248590000	-0.290338000	-4.229685000
1	-2.033430000	0.016405000	4.920817000
1	5.096551000	0.322956000	-0.705647000
1	0.639959000	0.300749000	5.232883000
1	4.772345000	0.417128000	1.979766000
1	-5.233123000	-0.706954000	0.807120000
1	1.864074000	-0.088796000	-4.835695000
1	-4.907136000	-0.817270000	-1.877284000
1	-0.807471000	-0.390539000	-5.147830000
6	-2.766488000	-0.264404000	2.173977000
6	2.607976000	0.080313000	-2.082756000
6	-2.181104000	-0.462822000	-2.642291000
6	2.024906000	0.257709000	2.733895000
1	-3.619198000	-0.308930000	2.842870000
1	3.458432000	0.145530000	-2.752835000
1	-2.847631000	-0.571995000	-3.491002000
1	2.690163000	0.377139000	3.582232000
8	-0.272391000	1.746578000	-0.053805000

$^{4}A_{2u}$:

26	-0.102447000	0.128189000	0.033271000
7	-0.322168000	-0.010868000	2.034437000
7	1.898226000	0.104242000	0.277458000
7	-2.049724000	-0.344590000	-0.184051000
7	0.163416000	-0.166818000	-1.944253000
6	-1.516004000	-0.051775000	2.740631000
6	2.862692000	0.171031000	-0.718130000
6	0.665188000	0.129001000	2.998768000
6	2.602081000	0.194548000	1.470525000
6	-3.017961000	-0.375185000	0.809829000
6	1.347247000	-0.030299000	-2.655387000
6	-2.744234000	-0.525294000	-1.372408000
6	-0.820925000	-0.336646000	-2.907235000
6	-1.270860000	0.057548000	4.156527000

6	4.177971000	0.288465000	-0.142512000
6	0.084409000	0.166185000	4.317030000
6	4.016321000	0.299305000	1.217139000
6	-4.322480000	-0.592990000	0.239353000
6	1.100279000	-0.121596000	-4.072086000
6	-4.152025000	-0.690061000	-1.115684000
6	-0.246120000	-0.315263000	-4.228322000
1	-2.035644000	0.043747000	4.918404000
1	5.095761000	0.351382000	-0.707529000
1	0.643914000	0.259416000	5.235589000
1	4.775966000	0.374296000	1.980337000
1	-5.239207000	-0.663001000	0.804861000
1	1.859479000	-0.054280000	-4.836680000
1	-4.902446000	-0.853534000	-1.874109000
1	-0.802560000	-0.436058000	-5.145550000
6	-2.771760000	-0.217696000	2.169080000
6	2.603760000	0.130525000	-2.083607000
6	-2.173242000	-0.514978000	-2.639623000
6	2.027604000	0.213363000	2.736138000
1	-3.625844000	-0.243263000	2.837400000
1	3.451677000	0.213145000	-2.755145000
1	-2.836581000	-0.651167000	-3.487085000
1	2.695420000	0.306678000	3.585888000
8	-0.278746000	1.743296000	-0.049399000

$^6A_{2u}$:

26	-0.116964000	0.267879000	0.027245000
7	-0.324983000	-0.019795000	2.070798000
7	1.957416000	0.087849000	0.285631000
7	-2.104841000	-0.370096000	-0.190205000
7	0.168146000	-0.178047000	-1.979040000
6	-1.520559000	-0.108790000	2.766368000
6	2.911325000	0.118960000	-0.716756000
6	0.670047000	0.140064000	3.022133000
6	2.643221000	0.208180000	1.481724000
6	-3.055090000	-0.436556000	0.814428000
6	1.364046000	-0.089132000	-2.674362000
6	-2.787987000	-0.518680000	-1.384877000
6	-0.827591000	-0.328329000	-2.931620000
6	-1.268769000	-0.003044000	4.184848000
6	4.228983000	0.248558000	-0.135185000
6	0.085299000	0.150883000	4.342984000
6	4.063115000	0.303883000	1.224982000
6	-4.364024000	-0.647367000	0.237257000
6	1.112224000	-0.186796000	-4.093222000
6	-4.198871000	-0.697414000	-1.123290000
6	-0.242639000	-0.333010000	-4.252349000
1	-2.024924000	-0.048981000	4.954045000
1	5.152529000	0.290411000	-0.692699000
1	0.637632000	0.254161000	5.264844000
1	4.826263000	0.399437000	1.982713000
1	-5.281630000	-0.744099000	0.798000000
1	1.870458000	-0.155121000	-4.861122000
1	-4.956898000	-0.841886000	-1.878365000
1	-0.793633000	-0.442476000	-5.174209000
6	-2.775454000	-0.303911000	2.178365000
6	2.625471000	0.046493000	-2.083690000
6	-2.189071000	-0.484781000	-2.648380000
6	2.037056000	0.243224000	2.741635000
1	-3.621668000	-0.366362000	2.855566000
1	3.473327000	0.093298000	-2.760096000
1	-2.849562000	-0.601326000	-3.502085000
1	2.698431000	0.353996000	3.595438000
8	-0.286970000	1.901541000	-0.061685000

$^4\Delta_{zz}$:

26	-0.103910000	0.139639000	0.032909000
7	-0.317957000	-0.035385000	2.019437000
7	1.885422000	0.090994000	0.276058000
7	-2.035460000	-0.350418000	-0.181539000
7	0.163989000	-0.192614000	-1.926680000
6	-1.520436000	-0.099114000	2.730507000
6	2.855985000	0.150746000	-0.728785000
6	0.675847000	0.139077000	2.987077000
6	2.587485000	0.230781000	1.477640000
6	-3.008020000	-0.392135000	0.822618000
6	1.361151000	-0.076911000	-2.640132000
6	-2.738315000	-0.484270000	-1.383517000
6	-0.833863000	-0.327725000	-2.896739000
6	-1.262302000	0.012905000	4.141389000
6	4.161323000	0.297146000	-0.141969000
6	0.085215000	0.158384000	4.298753000
6	3.996682000	0.345471000	1.211706000
6	-4.308288000	-0.584565000	0.238212000
6	1.101009000	-0.166879000	-4.052238000
6	-4.142841000	-0.641377000	-1.115075000
6	-0.245633000	-0.320830000	-4.209669000
1	-2.025557000	-0.022601000	4.903988000
1	5.079291000	0.352389000	-0.707329000
1	0.644490000	0.266454000	5.215547000
1	4.752972000	0.448395000	1.975132000
1	-5.223333000	-0.666151000	0.805092000
1	1.863599000	-0.121507000	-4.814972000
1	-4.895580000	-0.778422000	-1.876605000
1	-0.804824000	-0.426270000	-5.126998000
6	-2.771897000	-0.267991000	2.176019000
6	2.614051000	0.079505000	-2.084991000
6	-2.183035000	-0.467423000	-2.646035000
6	2.027177000	0.259210000	2.737674000
1	-3.622129000	-0.312975000	2.845679000
1	3.462243000	0.143876000	-2.755661000
1	-2.848279000	-0.575548000	-3.494034000
1	2.690923000	0.378960000	3.585322000
8	-0.278226000	1.787178000	-0.053095000

$^4\Delta_{xy}$:

26	-0.121384000	0.303413000	0.024472000
7	-0.322354000	-0.042118000	2.074837000
7	1.936799000	0.068922000	0.283692000
7	-2.080355000	-0.385497000	-0.187006000
7	0.170701000	-0.197177000	-1.981846000
6	-1.516956000	-0.136417000	2.766896000
6	2.889789000	0.096531000	-0.720209000
6	0.673049000	0.113539000	3.022978000
6	2.621210000	0.191580000	1.481025000
6	-3.029539000	-0.452348000	0.819196000
6	1.366722000	-0.115238000	-2.673346000
6	-2.762306000	-0.532518000	-1.383081000
6	-0.824711000	-0.351639000	-2.930697000
6	-1.260878000	-0.031243000	4.208527000
6	4.225734000	0.225222000	-0.128091000
6	0.078659000	0.121709000	4.365309000
6	4.061318000	0.285540000	1.218158000
6	-4.356084000	-0.668375000	0.231638000
6	1.110846000	-0.218476000	-4.114938000
6	-4.192719000	-0.716692000	-1.115303000
6	-0.229807000	-0.359924000	-4.272766000
1	-2.022267000	-0.075544000	4.972529000
1	5.146121000	0.263670000	-0.690971000
1	0.636253000	0.228716000	5.283691000
1	4.819680000	0.383572000	1.980215000
1	-5.270458000	-0.764553000	0.797678000
1	1.873964000	-0.186680000	-4.877861000
1	-4.946143000	-0.860048000	-1.875016000
1	-0.786799000	-0.467270000	-5.191424000
6	-2.768132000	-0.329155000	2.183440000
6	2.624037000	0.017900000	-2.086858000
6	-2.181425000	-0.506868000	-2.650644000
6	2.034921000	0.220912000	2.745718000
1	-3.618050000	-0.396512000	2.853734000
1	3.477134000	0.056955000	-2.755373000
1	-2.847323000	-0.628643000	-3.497945000

1	2.702492000	0.328070000	3.593698000
8	-0.304127000	1.962255000	-0.050453000

UB3LYP/BS2 optimized structures of [Fe^{IV}(O)($Por^{+•}$)]$^{+}$:

$^{2}A_{1U}$:

26	-0.104137718	0.142270073	0.033080591
7	-0.317126903	-0.035454178	2.017444298
7	1.884075130	0.095784451	0.276767720
7	-2.035674735	-0.344334042	-0.182723810
7	0.163118771	-0.190193541	-1.924671036
6	-1.495872221	-0.099843616	2.714757220
6	2.837596251	0.156372480	-0.712839795
6	0.661436837	0.128241330	2.969870147
6	2.573807475	0.226678564	1.454606204
6	-2.991495523	-0.383782070	0.805707167
6	1.336937640	-0.080059192	-2.624434011
6	-2.724710754	-0.481996234	-1.360241882
6	-0.817539751	-0.334355502	-2.878068926
6	-1.253162647	0.009229166	4.144952913
6	4.163914524	0.308240540	-0.133732682
6	0.077315285	0.147903111	4.302662735
6	4.001266546	0.348955672	1.202780923
6	-4.313657250	-0.574626244	0.228618105
6	1.092437819	-0.172538890	-4.055517441
6	-4.148606256	-0.637579901	-1.106735111
6	-0.235928829	-0.331076830	-4.212116966
1	-2.023219008	-0.022835284	4.902316667
1	5.079259913	0.370095153	-0.704666422
1	0.641262506	0.254535438	5.218217177
1	4.753437083	0.451895537	1.971713663
1	-5.227727721	-0.648461955	0.800171019
1	1.860490887	-0.121754710	-4.813887650
1	-4.897129720	-0.774414996	-1.873939232
1	-0.799716701	-0.439705823	-5.127534645
6	-2.754660414	-0.261561991	2.159547582
6	2.595382966	0.084780484	-2.069322428
6	-2.167513567	-0.473123947	-2.628508705
6	2.013298670	0.249163602	2.721275412
1	-3.603902389	-0.301428201	2.830421282
1	3.441329019	0.155968409	-2.741765552
1	-2.833223356	-0.582141686	-3.475751276
1	2.677958373	0.368033819	3.568015329
8	-0.272172340	1.739312368	-0.051585953

$^{4}A_{1u}$:

26	-0.105016286	0.151949482	0.032292800
7	-0.318522801	-0.010939953	2.017258470
7	1.880439942	0.097006175	0.276550028
7	-2.030864018	-0.347098137	-0.182977815
7	0.160127042	-0.161640879	-1.926887313
6	-1.502016493	-0.039489256	2.712170427
6	2.832194815	0.174090144	-0.711287570
6	0.660222060	0.124284830	2.970953948
6	2.576708482	0.180962536	1.458591107
6	-2.988657512	-0.366035279	0.802054769
6	1.331182984	-0.015602697	-2.628309647
6	-2.716346050	-0.531604511	-1.359359654
6	-0.814644395	-0.336508869	-2.878817102
6	-1.259299093	0.078172065	4.142123297
6	4.162542016	0.282026075	-0.132889978
6	0.074866558	0.172475470	4.302052850
6	4.005327011	0.279527517	1.205033853
6	-4.306213039	-0.595869275	0.229993051
6	1.084916611	-0.101518705	-4.059844701
6	-4.137119698	-0.705807684	-1.101956772
6	-0.237521785	-0.306250387	-4.213898404
1	-2.031810108	0.077300412	4.897722950
1	5.076861903	0.346286638	-0.705255113
1	0.640507338	0.267006689	5.217926418
1	4.761863163	0.342007088	1.974088656
1	-5.220405088	-0.661672878	0.802386579
1	1.848881745	-0.020299178	-4.819761889
1	-4.881718643	-0.881477580	-1.865101003
1	-0.799916203	-0.430016289	-5.128280850
6	-2.757401225	-0.198363789	2.154565894
6	2.586087215	0.148258949	-2.071374266
6	-2.160166210	-0.526075221	-2.625282157
6	2.018250690	0.199145875	2.723171365
1	-3.610907589	-0.215603419	2.821221225
1	3.431878335	0.238116323	-2.741999510
1	-2.821743416	-0.668438122	-3.470976450
1	2.685779156	0.287210691	3.571588140
8	-0.283736501	1.751406703	-0.041849555

$^{2}A_{2u}$:

26	-0.103312209	0.135496126	0.033084565
7	-0.321578073	-0.013341923	2.031802126
7	1.896536433	0.107529751	0.277563203
7	-2.049305400	-0.339425908	-0.184623772
7	0.163316620	-0.168680040	-1.941339988
6	-1.498704725	-0.062530494	2.730557616
6	2.851432235	0.169643333	-0.708376703
6	0.655901293	0.128524599	2.986294948
6	2.591989391	0.202994269	1.454397159
6	-3.006954850	-0.375185312	0.799924862
6	1.332386144	-0.042676493	-2.644094857
6	-2.736704237	-0.511554389	-1.357473428
6	-0.811583909	-0.334498099	-2.894719490
6	-1.263546371	0.041749792	4.141646350
6	4.163549628	0.291336247	-0.141504026
6	0.084364583	0.158180601	4.302072301
6	4.001263870	0.308780139	1.211252111
6	-4.309510929	-0.587680597	0.237788287
6	1.096284983	-0.137130395	-4.055660586
6	-4.139752396	-0.676339164	-1.111266303
6	-0.244347201	-0.322497501	-4.212556630
1	-2.030337397	0.020039950	4.902651079
1	5.081860780	0.351433978	-0.707756009
1	0.645074026	0.252335781	5.220951810
1	4.759648596	0.387525801	1.976895437
1	-5.226357336	-0.661437730	0.804810622
1	1.859040577	-0.077037081	-4.818653914
1	-4.889603143	-0.836060540	-1.872683507
1	-0.801969302	-0.444107454	-5.130081955
6	-2.756682036	-0.227664800	2.156457895
6	2.590686791	0.119998996	-2.069977042
6	-2.162023865	-0.502394465	-2.625995662
6	2.014339181	0.221329564	2.721522834
1	-3.608947014	-0.258423048	2.825953818
1	3.437820844	0.198223329	-2.742132996
1	-2.825940701	-0.633782697	-3.473038491
1	2.681782899	0.319297142	3.570318296
8	-0.275507106	1.727844859	-0.048830329

$^{4}A_{2u}$:

26	-0.103360774	0.135960311	0.033060581
7	-0.321105256	-0.015085333	2.031679442
7	1.896577190	0.106336697	0.277174990
7	-2.049091284	-0.340685362	-0.184061424
7	0.163345155	-0.171294102	-1.940940915
6	-1.500784599	-0.061787143	2.731016144
6	2.850565311	0.171824452	-0.706686062
6	0.654453949	0.124479968	2.985684757
6	2.592619018	0.202316344	1.456324611
6	-3.006590243	-0.372695114	0.798027529
6	1.334347163	-0.042147820	-2.644586847
6	-2.737083634	-0.513179048	-1.359231465
6	-0.809304590	-0.338144521	-2.893631549
6	-1.264157841	0.042102469	4.142223011
6	4.162709930	0.295909493	-0.141016308
6	0.083985147	0.155073045	4.301653692
6	4.001726918	0.311606318	1.211749428

6	-4.309674804	-0.582952250	0.236863468
6	1.096774876	-0.136485476	-4.056240301
6	-4.140742122	-0.674224382	-1.111949643
6	-0.243413167	-0.324916602	-4.211787897
1	-2.030473561	0.022467054	4.903759855
1	5.080300069	0.359071090	-0.708088777
1	0.645549252	0.247701442	5.220152928
1	4.760630932	0.391608097	1.976754073
1	-5.226492303	-0.653221864	0.804358599
1	1.858587127	-0.074150671	-4.819996741
1	-4.891317445	-0.833095643	-1.872834467
1	-0.801740683	-0.447082060	-5.128803064
6	-2.755786305	-0.224312516	2.157478878
6	2.589211527	0.122260760	-2.071262445
6	-2.162669058	-0.506659290	-2.624364272
6	2.015857935	0.217552115	2.720369319
1	-3.608764109	-0.252946410	2.826211430
1	3.436628829	0.202610532	-2.742851558
1	-2.825759061	-0.639143639	-3.471912949
1	2.682771514	0.314233754	3.569766747
8	-0.275607002	1.728493549	-0.049354018

$^{6}A_{2u}$:

26	-0.115820473	0.251362987	0.026801482
7	-0.323960474	0.010842219	2.067998774
7	1.951190585	0.057259280	0.288425184
7	-2.092056021	-0.399435645	-0.191295811
7	0.161616665	-0.148038613	-1.981417378
6	-1.509100715	-0.091359957	2.757789962
6	2.895078827	0.094714253	-0.704624716
6	0.663304888	0.134357589	3.015771187
6	2.634397644	0.162963248	1.471188601
6	-3.033825204	-0.457023935	0.802834291
6	1.349386769	-0.071701455	-2.670000412
6	-2.769366964	-0.560590326	-1.371219664
6	-0.819033768	-0.333680231	-2.925834143
6	-1.264608134	-0.005115855	4.168098402
6	4.211410563	0.208510972	-0.132483791
6	0.086070815	0.134264226	4.328690405
6	4.049655251	0.250053976	1.219604874
6	-4.338595520	-0.679875871	0.236322015
6	1.107784473	-0.186162829	-4.078862269
6	-4.174325319	-0.745129232	-1.114539706
6	-0.240244804	-0.349782886	-4.238048030
1	-2.021513247	-0.053959038	4.937997578
1	5.134202341	0.254893608	-0.693079251
1	0.637501210	0.221183948	5.254121069
1	4.814541048	0.337402769	1.978094061
1	-5.256894355	-0.769343430	0.799050854
1	1.866646030	-0.155786283	-4.847784095
1	-4.932378131	-0.897717802	-1.869570474
1	-0.787466747	-0.477492397	-5.161234569
6	-2.758566143	-0.302122150	2.164354098
6	2.608651421	0.046414526	-2.071780507
6	-2.175058201	-0.521221671	-2.635459701
6	2.031487300	0.206521556	2.731255751
1	-3.606063802	-0.365953895	2.838674557
1	3.458272249	0.090933369	-2.744993332
1	-2.835096137	-0.659422710	-3.485309826
1	2.696597930	0.297821594	3.583475974
8	-0.320659538	2.126562505	-0.053741288

UB3LYP-D3 optimized structures of $[Fe^{IV}(O)(Por^{+\bullet})]^{+}$:

$^{2}A_{1u}$:

26	-0.104137718	0.142270073	0.033080591
7	-0.317126903	-0.035454178	2.017444298
7	1.884075130	0.095784451	0.276767720
7	-2.035674735	-0.344334042	-0.182723810
7	0.163118771	-0.190193541	-1.924671036
6	-1.495872221	-0.099843616	2.714757220
6	2.837596251	0.156372480	-0.712839795
6	0.661436837	0.128241330	2.969870147
6	2.573807475	0.226678564	1.454606204
6	-2.991495523	-0.383782070	0.805707167
6	1.336937640	-0.080059192	-2.624434011
6	-2.724710754	-0.481996234	-1.360241882
6	-0.817539751	-0.334355502	-2.878068926
6	-1.253162647	0.009229166	4.144952913
6	4.163914524	0.308240540	-0.133732682
6	0.077315285	0.147903111	4.302662735
6	4.001266546	0.348955672	1.202780923
6	-4.313657250	-0.574626244	0.228618105
6	1.092437819	-0.172538890	-4.055517441
6	-4.148606256	-0.637579901	-1.106735111
6	-0.235928829	-0.331076830	-4.212116966
1	-2.023219008	-0.022835284	4.902316667
1	5.079259913	0.370095153	-0.704666422
1	0.641262506	0.254535438	5.218217177
1	4.753437083	0.451895537	1.971713663
1	-5.227727721	-0.648461955	0.800171019
1	1.860490887	-0.121754710	-4.813887650
1	-4.897129720	-0.774414996	-1.873939232
1	-0.799716701	-0.439705823	-5.127534645
6	-2.754660414	-0.261561991	2.159547582
6	2.595382966	0.084780484	-2.069322428
6	-2.167513567	-0.473123947	-2.628508705
6	2.013298670	0.249163602	2.721275412
1	-3.603902389	-0.301428201	2.830421282
1	3.441329019	0.155968409	-2.741765552
1	-2.833223356	-0.582141686	-3.475751276
1	2.677958373	0.368033819	3.568015329
8	-0.272172340	1.739312368	-0.051585953

$^{4}A_{1u}$:

26	-0.102510279	0.130834346	0.033876603
7	-0.317670380	-0.036320699	2.016963876
7	1.885331306	0.085004985	0.277729741
7	-2.033789212	-0.355652528	-0.179521170
7	0.167322391	-0.216201473	-1.922036345
6	-1.501819582	-0.065101336	2.713541966
6	2.834297344	0.181456662	-0.711309303
6	0.662114787	0.106458616	2.969875511
6	2.577099595	0.199066026	1.458980887
6	-2.992126018	-0.366454638	0.804665425
6	1.338244944	-0.066475324	-2.623921799
6	-2.722027525	-0.502623704	-1.359748587
6	-0.812547257	-0.358032256	-2.874163715
6	-1.257312555	0.055415877	4.142389670
6	4.160075724	0.338360397	-0.133406765
6	0.077207746	0.153133233	4.300968942
6	4.002557315	0.340454534	1.204807148
6	-4.313809791	-0.561961955	0.228978120
6	1.089337222	-0.140850369	-4.055505036
6	-4.147250450	-0.646142849	-1.105382075
6	-0.236722737	-0.322579695	-4.209781980
1	-2.029324758	0.054901832	4.898386293
1	5.071755112	0.426245839	-0.706610374
1	0.643553943	0.251705130	5.215872101
1	4.756102112	0.431269605	1.973917652
1	-5.228244002	-0.624328953	0.801239832
1	1.853167324	-0.059371181	-4.815321323
1	-4.894867485	-0.791362091	-1.871871085
1	-0.803492288	-0.422358452	-5.124384976
6	-2.759149473	-0.212670757	2.158671214
6	2.590179533	0.128753000	-2.070789136
6	-2.164690407	-0.501670619	-2.624742263
6	2.018636214	0.204965641	2.723717154
1	-3.611977509	-0.222871111	2.826068721
1	3.432291294	0.235652191	-2.743309929
1	-2.829601055	-0.608256538	-3.472978921
1	2.683356032	0.308456231	3.572497820
8	-0.267060003	1.725266049	-0.090126312

$^{2}A_{2u}$:

26	-0.103312209	0.135496126	0.033084565
7	-0.321578073	-0.013341923	2.031802126
7	1.896536433	0.107529751	0.277563203
7	-2.049305400	-0.339425908	-0.184623772
7	0.163316620	-0.168680040	-1.941339988
6	-1.498704725	-0.062530494	2.730557616
6	2.851432235	0.169643333	-0.708376703
6	0.655901293	0.128524599	2.986294948
6	2.591989391	0.202994269	1.454397159
6	-3.006954850	-0.375185312	0.799924862
6	1.332386144	-0.042676493	-2.644094857
6	-2.736704237	-0.511554389	-1.357473428
6	-0.811583909	-0.334498099	-2.894719490
6	-1.263546371	0.041749792	4.141646350
6	4.163549628	0.291336247	-0.141504026
6	0.084364583	0.158180601	4.302072301
6	4.001263870	0.308780139	1.211252111
6	-4.309510929	-0.587680597	0.237788287
6	1.096284983	-0.137130395	-4.055660586
6	-4.139752396	-0.676339164	-1.111266303
6	-0.244347201	-0.322497501	-4.212556630
1	-2.030337397	0.020039950	4.902651079
1	5.081860780	0.351433978	-0.707756009
1	0.645074026	0.252335781	5.220951810
1	4.759648596	0.387525801	1.976895437
1	-5.226357336	-0.661437730	0.804810622
1	1.859040577	-0.077037081	-4.818653914
1	-4.889603143	-0.836060540	-1.872683507
1	-0.801969302	-0.444107454	-5.130081955
6	-2.756682036	-0.227664800	2.156457895
6	2.590686791	0.119998996	-2.069977042
6	-2.162023865	-0.502394465	-2.625995662
6	2.014339181	0.221329564	2.721522834
1	-3.608947014	-0.258423048	2.825953818
1	3.437820844	0.198223329	-2.742132996
1	-2.825940701	-0.633782697	-3.473038491
1	2.681782899	0.319297142	3.570318296
8	-0.275507106	1.727844859	-0.048830329

$^{4}A_{2u}$:

26	-0.103360774	0.135960311	0.033060581
7	-0.321105256	-0.015085333	2.031679442
7	1.896577190	0.106336697	0.277174990
7	-2.049091284	-0.340685362	-0.184061424
7	0.163345155	-0.171294102	-1.940940915
6	-1.500784599	-0.061787143	2.731016144
6	2.850565311	0.171824452	-0.706686062
6	0.654453949	0.124479968	2.985684757
6	2.592619018	0.202316344	1.456324611
6	-3.006590243	-0.372695114	0.798027529
6	1.334347163	-0.042147820	-2.644586847
6	-2.737083634	-0.513179048	-1.359231465
6	-0.809304590	-0.338144521	-2.893631549
6	-1.264157841	0.042102469	4.142223011
6	4.162709930	0.295909493	-0.141016308
6	0.083985147	0.155073045	4.301653692
6	4.001726918	0.311606318	1.211749428
6	-4.309674804	-0.582952250	0.236863468
6	1.096774876	-0.136485476	-4.056240301
6	-4.140742122	-0.674224382	-1.111949643
6	-0.243413167	-0.324916602	-4.211787897
1	-2.030473561	0.022467054	4.903759855
1	5.080300069	0.359071090	-0.708088777
1	0.645549252	0.247701442	5.220152928
1	4.760630932	0.391608097	1.976754073
1	-5.226492303	-0.653221864	0.804358599
1	1.858587127	-0.074150671	-4.819996741
1	-4.891317445	-0.833095643	-1.872834467
1	-0.801740683	-0.447082060	-5.128803064
6	-2.755786305	-0.224312516	2.157478878
6	2.589211527	0.122260760	-2.071262445
6	-2.162669058	-0.506659290	-2.624364272
6	2.015857935	0.217552115	2.720369319
1	-3.608764109	-0.252946410	2.826211430
1	3.436628829	0.202610532	-2.742851558
1	-2.825759061	-0.639143639	-3.471912949
1	2.682771514	0.314233754	3.569766747
8	-0.275607002	1.728493549	-0.049354018

$^{6}A_{2u}$:

26	-0.116158881	0.255837971	0.026552464
7	-0.324150153	0.008951446	2.067473213
7	1.949927217	0.056581641	0.287881219
7	-2.090868620	-0.399198987	-0.190778641
7	0.161997288	-0.149457449	-1.980631495
6	-1.509658218	-0.094093124	2.757174784
6	2.893798424	0.095156162	-0.705617755
6	0.663892400	0.132704603	3.014884867
6	2.632467239	0.164284918	1.471300230
6	-3.032707260	-0.455939285	0.803747508
6	1.350313434	-0.074077043	-2.669008325
6	-2.767938316	-0.558713204	-1.371450680
6	-0.819539558	-0.335099492	-2.924738821
6	-1.264638475	-0.009758415	4.167783657
6	4.210163824	0.210277962	-0.133133790
6	0.086948654	0.130346184	4.328226521
6	4.047968298	0.252386177	1.219898954
6	-4.337780672	-0.677910927	0.236899076
6	1.108628515	-0.190621171	-4.077954602
6	-4.173276640	-0.742711919	-1.114952380
6	-0.240485823	-0.353706773	-4.237073205
1	-2.022087990	-0.060553328	4.936940595
1	5.132377752	0.256718884	-0.694564533
1	0.639726154	0.215997680	5.252903989
1	4.811757342	0.340337451	1.979349864
1	-5.255432918	-0.767315439	0.800602569
1	1.868429511	-0.162066624	-4.845941570
1	-4.930314986	-0.894875538	-1.870990653
1	-0.788747354	-0.482907470	-5.159381831
6	-2.759363836	-0.303179158	2.165271785
6	2.609613514	0.045378455	-2.072548945
6	-2.175469913	-0.520850307	-2.635902673
6	2.031596050	0.206812651	2.731659199
1	-3.607138298	-0.367288135	2.839225847
1	3.459452107	0.089881629	-2.745496594
1	-2.835873715	-0.658594795	-3.485548260
1	2.697055538	0.298835917	3.583542157
8	-0.319651980	2.125117404	-0.054207363

UB3LYP/BS2 optimized geometries of epoxidation reactant complexes between [Fe^{IV}(O)(Por$^{+\bullet}$)]$^{+}$ with substrates:

$^{4}Re_{1}$:

26	-0.073869000	0.084049000	0.007613000
7	-0.065813000	-0.054984000	2.013980000
7	1.935236000	-0.002209000	0.027345000
7	-2.037496000	-0.356710000	0.018111000
7	-0.036028000	-0.296338000	-1.969513000
6	-1.153256000	-0.108238000	2.848747000
6	2.788876000	-0.006348000	-1.090342000
6	1.043430000	0.105702000	2.861253000
6	2.762090000	0.143396000	1.112021000
6	-2.889768000	-0.372149000	1.136749000
6	1.051311000	-0.262609000	-2.802834000
6	-2.864364000	-0.493751000	-1.066951000
6	-1.148650000	-0.440916000	-2.819506000
6	-0.749422000	0.003833000	4.238646000
6	4.170248000	0.127941000	-0.656083000
6	0.604275000	0.137630000	4.247690000
6	4.152764000	0.219942000	0.700843000
6	-4.268627000	-0.537723000	0.703610000

6	0.648755000	-0.397716000	-4.192780000
6	-4.251760000	-0.613898000	-0.654165000
6	-0.706660000	-0.505029000	-4.203730000
1	-1.427459000	-0.021438000	5.078177000
1	5.021837000	0.143076000	-1.319670000
1	1.263358000	0.243825000	5.096268000
1	4.987014000	0.326560000	1.377567000
1	-5.117201000	-0.590147000	1.369161000
1	1.330298000	-0.408900000	-5.029923000
1	-5.083656000	-0.740990000	-1.330243000
1	-1.364734000	-0.623350000	-5.051498000
6	-2.489015000	-0.253727000	2.438543000
6	2.387249000	-0.121560000	-2.392500000
6	-2.449951000	-0.525186000	-2.409392000
6	2.344670000	0.204171000	2.451632000
1	-3.247800000	-0.278564000	3.212048000
1	3.147337000	-0.110331000	-3.165188000
1	-3.218872000	-0.635932000	-3.165286000
1	3.111601000	0.325780000	3.207793000
8	-0.221451000	1.705721000	-0.079065000
6	-0.964343000	4.191534000	-0.809717000
1	-1.414021000	4.342922000	0.166287000
1	-1.652091000	4.051359000	-1.638807000
6	0.363865000	4.180959000	-0.982232000
1	0.813971000	4.030849000	-1.959547000
1	1.050252000	4.324833000	-0.153022000
6	-0.853584000	4.275529000	-1.383982000
1	-0.168826000	3.605397000	-1.915331000
1	-1.841596000	4.214025000	-1.852568000
1	-0.475562000	5.297587000	-1.526355000

$^4Re_2$:

26	0.272537000	0.166521000	-0.129297000
7	0.146303000	0.018811000	1.868774000
7	2.257424000	-0.112961000	0.006871000
7	-1.718066000	-0.059426000	-0.238358000
7	0.393117000	-0.215715000	-2.100045000
6	-1.022601000	0.133992000	2.642671000
6	3.164401000	-0.142439000	-1.027747000
6	1.178057000	0.001805000	2.775156000
6	3.031907000	-0.105551000	1.175649000
6	-2.620360000	0.083668000	0.784370000
6	1.561441000	-0.235685000	-2.878218000
6	-2.497036000	-0.166234000	-1.405828000
6	-0.641930000	-0.307319000	-3.000623000
6	-0.679535000	0.170250000	4.053393000
6	4.524127000	-0.162558000	-0.525171000
6	0.675505000	0.081424000	4.135020000
6	4.444066000	-0.145580000	0.833636000
6	-3.985101000	0.054790000	0.283099000
6	1.215976000	-0.352502000	-4.285933000
6	-3.909216000	-0.106526000	-1.064152000
6	-0.141664000	-0.404446000	-4.359152000
1	-1.397583000	0.244790000	4.856273000
1	5.407004000	-0.194323000	-1.145457000
1	1.295690000	0.071426000	5.018595000
1	5.248106000	-0.158260000	1.554220000
1	-4.866123000	0.138462000	0.901334000
1	1.934668000	-0.396565000	-5.090552000
1	-4.714768000	-0.180246000	-1.779248000
1	-0.763909000	-0.497634000	-5.236291000
6	-2.295981000	0.190463000	2.145921000
6	2.839746000	-0.183220000	-2.390910000
6	-2.006533000	-0.290760000	-2.676424000
6	2.540510000	-0.060383000	2.453407000
1	-3.115615000	0.291035000	2.847951000
1	3.659365000	-0.202814000	-3.099759000
1	-2.714651000	-0.368415000	-3.493225000
1	3.248430000	-0.063478000	3.274101000
8	0.277958000	1.795683000	-0.210239000
6	-2.048593000	3.742351000	0.774365000
1	-2.040906000	3.572645000	1.846569000
1	-3.022747000	3.771545000	0.292356000
6	-0.907425000	3.934661000	0.077172000
1	0.039609000	3.955671000	0.609292000

$^4Re_3$:

26	0.034537000	1.375821000	-0.211480000
7	-0.076880000	1.534307000	1.785008000
7	2.033678000	1.293840000	-0.059447000
7	-1.925694000	0.950363000	-0.253668000
7	0.184720000	0.710669000	-2.098317000
6	-1.218194000	1.616054000	2.551191000
6	2.948918000	1.142387000	-1.109712000
6	0.978111000	1.813205000	2.664249000
6	2.797245000	1.598772000	1.045254000
6	-2.842056000	1.114504000	0.793873000
6	1.325833000	0.632847000	-2.865316000
6	-2.690154000	0.657453000	-1.361053000
6	-0.871252000	0.445775000	-2.980652000
6	-0.891474000	1.931219000	3.936212000
6	4.308352000	1.345809000	-0.618894000
6	0.457477000	2.054093000	4.006301000
6	4.214221000	1.625253000	0.704930000
6	-4.200339000	0.897174000	0.305989000
6	1.000668000	0.297427000	-4.245940000
6	-4.105980000	0.615925000	-1.017469000
6	-0.348970000	0.183641000	-4.318074000
1	-1.619309000	2.037735000	4.726235000
1	5.194794000	1.274759000	-1.231042000
1	1.069427000	2.281435000	4.866204000
1	5.007099000	1.832920000	1.407359000
1	-5.085979000	0.956982000	0.920504000
1	1.729939000	0.172180000	-5.031882000
1	-4.898008000	0.397206000	-1.717511000
1	-0.959962000	-0.055734000	-5.175394000
6	-2.523580000	1.425721000	2.090052000
6	2.629776000	0.840950000	-2.408047000
6	-2.200957000	0.424516000	-2.649096000
6	2.306610000	1.849823000	2.329373000
1	-3.329953000	1.525820000	2.806630000
1	3.435319000	0.752282000	-3.127047000
1	-2.920681000	0.207828000	-3.429350000
1	3.025204000	2.081035000	3.106484000
8	-0.084270000	2.958712000	-0.553235000
6	-0.282029000	6.390459000	-0.852327000
1	-0.360535000	5.882562000	0.104298000
1	-0.362860000	5.757226000	-1.732847000
6	-0.094211000	7.712458000	-0.946628000
1	-0.023356000	8.308219000	-0.034148000
6	0.013765000	8.488715000	-2.235566000
1	0.999489000	8.976631000	-2.286173000
1	-0.037333000	7.797534000	-3.087483000
6	-1.078954000	9.570499000	-2.373646000
1	-1.038636000	10.280749000	-1.538791000
1	-0.953628000	10.137955000	-3.302500000
1	-2.077097000	9.118411000	-2.380283000

$^4Re_4$:

26	0.354875000	0.215175000	0.026306000
7	0.260280000	-0.153201000	1.991030000
7	2.318194000	-0.159314000	0.089018000
7	-1.639159000	0.077720000	-0.061484000
7	0.418020000	0.051364000	-1.969268000
6	-0.870063000	-0.219396000	2.784194000
6	3.201344000	-0.222072000	-0.990468000
6	1.334719000	-0.174653000	2.882130000
6	3.118468000	-0.177847000	1.214653000
6	-2.532452000	-0.040933000	1.005861000
6	1.534270000	-0.045080000	-2.768915000
6	-2.427953000	0.279046000	-1.174986000
6	-0.653743000	0.244922000	-2.854626000

6	-0.508435000	-0.302530000	4.188148000
6	4.571718000	-0.303525000	-0.510293000
6	0.849088000	-0.262795000	4.250538000
6	4.519574000	-0.265380000	0.848404000
6	-3.902965000	0.054565000	0.522276000
6	1.174998000	0.064673000	-4.171169000
6	-3.835774000	0.264428000	-0.819275000
6	-0.170993000	0.255272000	-4.223493000
1	-1.220475000	-0.376679000	4.996667000
1	5.437975000	-0.374365000	-1.151517000
1	1.487156000	-0.300778000	5.121142000
1	5.333328000	-0.302562000	1.557323000
1	-4.777059000	-0.030804000	1.150966000
1	1.880685000	0.005341000	-4.986531000
1	-4.643720000	0.384430000	-1.525791000
1	-0.802033000	0.379752000	-5.091137000
6	-2.183284000	-0.189527000	2.326259000
6	2.845432000	-0.184901000	-2.315142000
6	-1.965691000	0.382295000	-2.489404000
6	2.662296000	-0.160502000	2.530093000
1	-2.976619000	-0.264951000	3.060422000
1	3.632012000	-0.244369000	-3.058108000
1	-2.701596000	0.534459000	-3.270220000
1	3.402072000	-0.177431000	3.321614000
8	0.448811000	1.838917000	0.090997000
6	-2.420968000	3.366223000	-0.818851000
1	-3.104609000	3.109679000	-1.627877000
6	-1.112639000	3.601325000	-1.110339000
1	-0.454223000	3.922140000	-0.308818000
6	-2.992580000	3.478722000	0.556745000
1	-3.837289000	4.182566000	0.582390000
1	-3.389653000	2.509618000	0.896971000
1	-2.234685000	3.808662000	1.275415000
6	-0.511336000	3.491654000	-2.471855000
1	-0.014628000	4.429763000	-2.758392000
1	0.264256000	2.712448000	-2.472512000
1	-1.258339000	3.244038000	-3.233394000

^{4}Re$_5$:

26	0.212906000	-0.825517000	0.163381000
7	0.277683000	-0.909486000	2.167944000
7	2.206919000	-1.055644000	0.124552000
7	-1.770200000	-1.130406000	0.250350000
7	0.159184000	-1.275313000	-1.793361000
6	-0.788662000	-0.853604000	3.039821000
6	3.020220000	-1.143463000	-1.014144000
6	1.418833000	-0.808807000	2.975227000
6	3.079854000	-0.937694000	1.184456000
6	-2.583319000	-1.042576000	1.388714000
6	1.225150000	-1.335426000	-2.664543000
6	-2.643214000	-1.250137000	-0.809611000
6	-0.982625000	-1.375146000	-2.601154000
6	-0.327420000	-0.732815000	4.412636000
6	4.422311000	-1.092069000	-0.628453000
6	1.030587000	-0.704154000	4.373813000
6	4.458180000	-0.966126000	0.724008000
6	-3.984888000	-1.125963000	1.006006000
6	0.764552000	-1.489507000	-4.034592000
6	-4.020697000	-1.254921000	-0.346215000
6	-0.593511000	-1.512835000	-3.996504000
1	-0.976815000	-0.682077000	5.273489000
1	5.248556000	-1.151549000	-1.320902000
1	1.725816000	-0.625539000	5.196131000
1	5.320082000	-0.900330000	1.370666000
1	-4.810473000	-1.093432000	1.701051000
1	1.414769000	-1.570735000	-4.892509000
1	-4.881904000	-1.349270000	-0.990245000
1	-1.288024000	-1.618002000	-4.816451000
6	-2.136515000	-0.909238000	2.678282000
6	2.572960000	-1.267174000	-2.304340000
6	-2.278480000	-1.358888000	-2.153561000
6	2.714736000	-0.814881000	2.526912000
1	-2.872366000	-0.853040000	3.471740000
1	3.308702000	-1.325480000	-3.097747000
1	-3.073682000	-1.445650000	-2.884540000
1	3.509830000	-0.728931000	3.258100000
8	0.181292000	0.793552000	0.014582000
6	-0.743367000	4.539801000	-1.746061000
1	-0.647842000	4.291435000	-2.801716000
6	-1.818282000	5.251932000	-1.326749000
1	-2.556790000	5.585819000	-2.053188000
6	-2.049550000	5.626480000	0.113065000
1	-1.869467000	6.708776000	0.233885000
1	-3.109528000	5.482915000	0.367663000
6	0.299388000	4.764166000	0.550627000
1	0.942869000	4.230687000	1.260049000
1	0.700294000	5.783643000	0.459107000
6	-1.146066000	4.832555000	1.079501000
1	-1.171670000	5.289597000	2.075662000
1	-1.536962000	3.810191000	1.184199000
6	0.347313000	4.067884000	-0.825484000
1	0.259632000	2.974120000	-0.689167000
1	1.325975000	4.222387000	-1.303377000

^{4}Re$_6$:

26	0.209263000	0.144992000	-0.336025000
7	0.256537000	0.340266000	1.668301000
7	2.150712000	-0.374259000	-0.295482000
7	-1.797766000	0.138245000	-0.250153000
7	0.098132000	-0.549222000	-2.215552000
6	-0.790264000	0.668173000	2.498816000
6	2.945436000	-0.732130000	-1.390506000
6	1.400825000	0.381142000	2.482515000
6	3.027812000	-0.249062000	0.762660000
6	-2.595285000	0.486136000	0.851173000
6	1.147904000	-0.872819000	-3.048017000
6	-2.676973000	-0.001937000	-1.302222000
6	-1.043235000	-0.593311000	-3.029254000
6	-0.321353000	0.910784000	3.850108000
6	4.333757000	-0.841623000	-0.981952000
6	1.028342000	0.734995000	3.840225000
6	4.382680000	-0.544285000	0.346593000
6	-3.990580000	0.538290000	0.455711000
6	0.676914000	-1.136158000	-4.392657000
6	-4.039087000	0.235883000	-0.872245000
6	-0.673841000	-0.963646000	-4.381821000
1	-0.958851000	1.169988000	4.681924000
1	5.145520000	-1.114942000	-1.639401000
1	1.723257000	0.821021000	4.662196000
1	5.242473000	-0.525164000	0.999108000
1	-4.809114000	0.757786000	1.125104000
1	1.311115000	-1.419010000	-5.219269000
1	-4.904841000	0.166802000	-1.513652000
1	-1.371867000	-1.077098000	-5.197737000
6	-2.134410000	0.745021000	2.116066000
6	2.488852000	-0.954871000	-2.665335000
6	-2.324590000	-0.337122000	-2.612759000
6	2.679355000	0.110957000	2.067952000
1	-2.856956000	1.008698000	2.879347000
1	3.211875000	-1.223564000	-3.426789000
1	-3.122310000	-0.409895000	-3.343026000
1	3.476645000	0.171891000	2.799683000
8	0.403183000	1.723611000	-0.697128000
6	-0.460084000	4.073775000	-0.505663000
1	0.223956000	3.982341000	-1.340607000
6	-1.793816000	4.157061000	-0.724664000
1	-2.181752000	4.090222000	-1.737867000
6	-2.798141000	4.311065000	0.378826000
1	-3.434605000	5.195030000	0.191249000
1	-3.515319000	3.466513000	0.341276000
6	-0.861569000	4.371558000	1.966070000
1	-0.472182000	4.467534000	2.976830000

6	-2.191941000	4.402088000	1.750647000
1	-2.878086000	4.522031000	2.585840000
6	0.145898000	4.240021000	0.858985000
1	0.826580000	3.397194000	1.059459000
1	0.809228000	5.125104000	0.858725000

$^{4}Re_{7}$:

26	-0.016601000	0.118405000	-0.345723000
7	-0.206178000	-1.117506000	1.227814000
7	0.221991000	-1.485758000	-1.531014000
7	-0.776523000	1.603027000	0.775652000
7	-0.350623000	1.234480000	-1.982523000
6	-0.463572000	-0.753384000	2.556290000
6	0.387204000	-1.494123000	-2.899429000
6	0.098510000	-2.461465000	1.272921000
6	0.479385000	-2.801203000	-1.120936000
6	-0.957328000	1.607946000	2.142760000
6	-0.109693000	0.866542000	-3.313570000
6	-1.028106000	2.919641000	0.366603000
6	-0.654933000	2.578416000	-2.027915000
6	-0.324795000	-1.910280000	3.423735000
6	0.736426000	-2.822207000	-3.368142000
6	0.020829000	-2.961775000	2.632601000
6	0.795225000	-3.627328000	-2.273135000
6	-1.339944000	2.928046000	2.606989000
6	-0.287269000	2.014321000	-4.185605000
6	-1.381809000	3.736998000	1.513943000
6	-0.622989000	3.067990000	-3.393290000
1	-0.483199000	-1.895868000	4.491696000
1	0.904627000	-3.081185000	-4.402621000
1	0.204791000	-3.986370000	2.918824000
1	1.020913000	-4.682137000	-2.225585000
1	-1.548628000	3.178021000	3.636279000
1	-0.170891000	1.990488000	-5.258757000
1	-1.632341000	4.785984000	1.463263000
1	-0.838127000	4.085300000	-3.683487000
6	-0.805825000	0.504247000	2.984462000
6	0.236692000	-0.389954000	-3.741068000
6	-0.966037000	3.376677000	-0.925359000
6	0.429978000	-3.255035000	0.172637000
1	-0.981198000	0.653088000	4.043551000
1	0.395416000	-0.542560000	-4.802243000
1	-1.185913000	4.421934000	-1.108452000
1	0.649796000	-4.300362000	0.355839000
8	1.557985000	0.476394000	-0.155351000
6	4.579208000	1.467471000	0.018031000
1	4.198075000	1.738135000	0.996444000
1	4.189631000	2.035997000	-0.817905000
6	5.479125000	0.473195000	-0.175392000
1	5.806923000	0.271557000	-1.193882000
6	6.089315000	-0.370629000	0.845650000
6	5.772770000	-0.268703000	2.225808000
6	6.394055000	-1.092089000	3.158780000
6	7.346368000	-2.040168000	2.744861000
6	7.671002000	-2.157894000	1.384188000
6	7.050421000	-1.334609000	0.448149000
1	5.040646000	0.458442000	2.558702000
1	6.146167000	-1.000411000	4.210860000
1	7.831358000	-2.677517000	3.476562000
1	8.407232000	-2.886645000	1.062944000
1	7.304506000	-1.421649000	-0.603992000

$^{4}Re_{8}$:

26	0.222312000	1.317321000	0.148566000
7	0.059111000	1.170981000	2.145182000
7	2.218457000	1.367638000	0.355192000
7	-1.685877000	0.715814000	-0.012201000
7	0.472368000	0.918027000	-1.800729000
6	-1.113952000	1.034804000	2.881967000
6	3.164427000	1.428130000	-0.663182000
6	1.058248000	1.403857000	3.085804000
6	2.933962000	1.573810000	1.530901000
6	-2.629403000	0.638698000	1.007904000
6	1.647635000	1.036658000	-2.536476000
6	-2.400780000	0.506265000	-1.187234000
6	-0.525484000	0.681682000	-2.741481000
6	-0.836720000	1.173857000	4.300466000
6	4.487197000	1.660144000	-0.110649000
6	0.498951000	1.401218000	4.425742000
6	4.345448000	1.750062000	1.238462000
6	-3.945056000	0.362464000	0.458694000
6	1.377078000	0.857050000	-3.951846000
6	-3.804562000	0.281581000	-0.891114000
6	0.040900000	0.639424000	-4.077923000
1	-1.580708000	1.090222000	5.078611000
1	5.389783000	1.733796000	-0.698556000
1	1.075358000	1.542302000	5.327883000
1	5.107923000	1.912542000	1.985442000
1	-4.840690000	0.244314000	1.049979000
1	2.129248000	0.890065000	-4.725748000
1	-4.561172000	0.083259000	-1.635381000
1	-0.528927000	0.457069000	-4.976719000
6	-2.369807000	0.792250000	2.356772000
6	2.903349000	1.280282000	-2.012544000
6	-1.866535000	0.497022000	-2.462200000
6	2.397847000	1.599015000	2.804633000
1	-3.200815000	0.705098000	3.046812000
1	3.737018000	1.347447000	-2.701553000
1	-2.538646000	0.318603000	-3.293254000
1	3.071988000	1.768280000	3.635988000
8	-0.035198000	2.923513000	0.026328000
6	-0.428450000	6.701946000	-2.650861000
6	-0.652004000	7.081513000	-1.225337000
6	-0.600503000	6.199642000	-0.181851000
1	-0.383554000	5.154890000	-0.401638000
1	-0.866421000	8.133211000	-1.040610000
1	0.411376000	7.268559000	-3.078878000
1	-1.304839000	6.955366000	-3.265032000
1	-0.221244000	5.634069000	-2.763872000
6	-0.806795000	6.507285000	1.218906000
6	-0.705230000	5.441389000	2.158732000
6	-0.891590000	5.671733000	3.517669000
6	-1.183970000	6.965999000	3.978147000
6	-1.289074000	8.032908000	3.064345000
6	-1.104103000	7.811774000	1.706355000
1	-1.188557000	8.643788000	1.016641000
1	-1.515256000	9.030426000	3.424542000
1	-1.329507000	7.147207000	5.037799000
1	-0.810678000	4.850624000	4.221943000
1	-0.479105000	4.446661000	1.787068000

UB3LYP/BS2 optimized geometries of epoxidation Transition states for the reaction of $[Fe^{IV}(O)(Por^{+\bullet})]^{+}$ with substrates:

$^{4}TS_{1}$:

26	-0.040890000	0.181714000	-0.093164000
7	-0.132215000	-0.058861000	1.903447000
7	1.971124000	0.033839000	0.028167000
7	-1.992857000	-0.222411000	-0.197321000
7	0.098750000	-0.169223000	-2.072947000
6	-1.277042000	-0.155098000	2.690226000
6	2.879761000	0.028790000	-1.020138000
6	0.915712000	0.069984000	2.810066000
6	2.746583000	0.164655000	1.175914000
6	-2.901953000	-0.285517000	0.853905000
6	1.258242000	-0.166051000	-2.854118000
6	-2.781343000	-0.282252000	-1.355789000
6	-0.943903000	-0.244680000	-2.982517000
6	-0.935918000	-0.107976000	4.090391000
6	4.226690000	0.146674000	-0.527511000
6	0.419964000	0.039646000	4.164421000
6	4.144077000	0.233739000	0.836922000
6	-4.252340000	-0.390067000	0.354345000

6	0.920238000	-0.255593000	-4.253070000
6	-4.177925000	-0.380795000	-1.008129000
6	-0.443268000	-0.302417000	-4.332772000
1	-1.647647000	-0.178315000	4.899185000
1	5.111219000	0.157900000	-1.146460000
1	1.038682000	0.111197000	5.046364000
1	4.947941000	0.330210000	1.551275000
1	-5.131286000	-0.470142000	0.976387000
1	1.639608000	-0.280706000	-5.057869000
1	-4.983513000	-0.452963000	-1.723467000
1	-1.060688000	-0.374990000	-5.215438000
6	-2.566883000	-0.258855000	2.199743000
6	2.546258000	-0.073844000	-2.366696000
6	-2.293708000	-0.282003000	-2.645077000
6	2.253912000	0.190750000	2.470886000
1	-3.375593000	-0.325557000	2.919181000
1	3.358594000	-0.077914000	-3.085029000
1	-3.011443000	-0.338379000	-3.456078000
1	2.969518000	0.291698000	3.279408000
8	-0.184242000	1.862252000	-0.135840000
6	-1.254771000	3.428690000	-0.338931000
1	-1.517086000	3.227531000	-1.372086000
6	-2.232724000	3.379815000	0.647350000
1	-3.206696000	2.940259000	0.459149000
1	-2.035859000	3.741375000	1.651077000
1	-0.375952000	4.046943000	-0.188070000

$^4TS_2$:

26	0.079393000	0.141467000	-0.089991000
7	-0.020245000	-0.140770000	1.898375000
7	2.089780000	0.025457000	0.037170000
7	-1.870891000	-0.328840000	-0.211779000
7	0.234593000	-0.168728000	-2.078330000
6	-1.169594000	-0.250484000	2.677447000
6	3.001090000	0.099176000	-1.011748000
6	1.027097000	-0.047487000	2.815473000
6	2.860968000	0.102482000	1.190125000
6	-2.784495000	-0.412985000	0.835994000
6	1.386257000	-0.072288000	-2.853426000
6	-2.643403000	-0.425161000	-1.370032000
6	-0.805720000	-0.283434000	-2.991931000
6	-0.834415000	-0.235136000	4.080319000
6	4.346064000	0.209228000	-0.505681000
6	0.522577000	-0.109346000	4.165467000
6	4.259162000	0.210494000	0.859188000
6	-4.125350000	-0.569042000	0.327993000
6	1.058957000	-0.141699000	-4.255196000
6	-4.038043000	-0.576389000	-1.034811000
6	-0.299648000	-0.273548000	-4.341039000
1	-1.551232000	-0.319513000	4.883384000
1	5.231646000	0.269760000	-1.120405000
1	1.137192000	-0.070237000	5.052350000
1	5.059372000	0.272405000	1.581407000
1	-5.006987000	-0.669033000	0.943249000
1	1.780102000	-0.101517000	-5.057814000
1	-4.834107000	-0.683460000	-1.756420000
1	-0.909272000	-0.362211000	-5.227745000
6	-2.457207000	-0.369945000	2.181362000
6	2.673314000	0.058756000	-2.357306000
6	-2.148366000	-0.398474000	-2.661011000
6	2.364739000	0.072242000	2.485533000
1	-3.266843000	-0.450809000	2.898295000
1	3.484541000	0.121185000	-3.074252000
1	-2.860064000	-0.486518000	-3.474495000
1	3.079480000	0.135377000	3.298635000
8	-0.081084000	1.799902000	-0.095725000
6	-1.097624000	3.494071000	0.527329000
1	-1.019679000	3.231048000	1.575131000
1	-2.014266000	3.214243000	0.019769000
6	-0.219384000	4.390826000	-0.047355000
1	0.641852000	4.715949000	0.533941000
6	-0.331197000	4.909881000	-1.437290000
1	0.548924000	4.617145000	-2.028484000
1	-1.227165000	4.546005000	-1.947176000
1	-0.344776000	6.009444000	-1.443167000

$^4TS_3$:

26	0.047540000	0.107268000	-0.131326000
7	0.018477000	0.165280000	1.885329000
7	2.063467000	0.185087000	-0.057716000
7	-1.853789000	-0.544173000	-0.096050000
7	0.185715000	-0.526938000	-2.035126000
6	-1.098962000	0.103199000	2.708899000
6	2.942699000	0.142383000	-1.132673000
6	1.068878000	0.515315000	2.728640000
6	2.848239000	0.532819000	1.036861000
6	-2.733184000	-0.519329000	0.986406000
6	1.311431000	-0.481691000	-2.857808000
6	-2.639854000	-0.906951000	-1.187884000
6	-0.860217000	-0.890756000	-2.878482000
6	-0.741222000	0.401599000	4.072851000
6	4.283253000	0.451035000	-0.703747000
6	0.601780000	0.657284000	4.085067000
6	4.224632000	0.693454000	0.641093000
6	-4.065200000	-0.875982000	0.562995000
6	0.959788000	-0.828014000	-4.212930000
6	-4.007641000	-1.115415000	-0.780094000
6	-0.382390000	-1.080919000	-4.225875000
1	-1.431694000	0.405705000	4.902913000
1	5.147376000	0.470182000	-1.350824000
1	1.227677000	0.911736000	4.927277000
1	5.031390000	0.949890000	1.311316000
1	-4.920587000	-0.940341000	1.218757000
1	1.658531000	-0.876964000	-5.034670000
1	-4.806298000	-1.414790000	-1.442193000
1	-1.000314000	-1.377841000	-5.060055000
6	-2.382734000	-0.211764000	2.288675000
6	2.590911000	-0.164464000	-2.439106000
6	-2.177566000	-1.061209000	-2.483766000
6	2.384495000	0.691656000	2.333016000
1	-3.166136000	-0.230815000	3.038247000
1	3.379150000	-0.167867000	-3.183842000
1	-2.895875000	-1.351383000	-3.242656000
1	3.106672000	0.961658000	3.095598000
8	-0.263586000	1.714107000	-0.437279000
6	-1.314693000	3.214426000	-1.407215000
1	-2.280526000	2.816001000	-1.120239000
1	-0.940488000	2.932145000	-2.385494000
6	-0.731531000	4.247607000	-0.700299000
1	-1.196324000	4.566175000	0.232343000
6	0.529585000	4.941925000	-1.090277000
1	1.301448000	4.699278000	-0.340920000
1	0.893781000	4.561803000	-2.051962000
6	0.372983000	6.482888000	-1.142268000
1	0.036515000	6.880625000	-0.178346000
1	1.332479000	6.949872000	-1.383984000
1	-0.353112000	6.779409000	-1.905828000

$^4TS_4$:

26	0.228686000	0.233661000	0.043661000
7	0.207363000	-0.174032000	2.008871000
7	2.210163000	-0.072622000	0.042159000
7	-1.755438000	0.009647000	0.012147000
7	0.242766000	0.100384000	-1.951167000
6	-0.896700000	-0.233956000	2.843135000
6	3.066928000	-0.032182000	-1.056085000
6	1.308539000	-0.219666000	2.864922000
6	3.046030000	-0.121478000	1.147814000
6	-2.617532000	-0.084378000	1.115486000
6	1.349091000	0.107873000	-2.790029000
6	-2.592166000	0.123727000	-1.083893000
6	-0.859051000	0.198629000	-2.809850000

6	-0.492698000	-0.338591000	4.222894000
6	4.442155000	-0.075784000	-0.623445000
6	0.873043000	-0.326321000	4.237980000
6	4.428265000	-0.126578000	0.742778000
6	-3.994242000	-0.046805000	0.677256000
6	0.941244000	0.200784000	-4.169943000
6	-3.977354000	0.082961000	-0.681726000
6	-0.422888000	0.260593000	-4.182545000
1	-1.176002000	-0.412738000	5.055868000
1	5.292781000	-0.067977000	-1.289047000
1	1.538828000	-0.388017000	5.086252000
1	5.264821000	-0.171968000	1.424278000
1	-4.845669000	-0.124055000	1.337447000
1	1.623406000	0.210642000	-5.007307000
1	-4.812995000	0.135424000	-1.364135000
1	-1.086914000	0.325574000	-5.032010000
6	-2.222663000	-0.196430000	2.425653000
6	2.669626000	0.047125000	-2.373982000
6	-2.173355000	0.231119000	-2.406600000
6	2.626345000	-0.183151000	2.470464000
1	-2.992425000	-0.260224000	3.186169000
1	3.440142000	0.065022000	-3.136304000
1	-2.938200000	0.309308000	-3.171277000
1	3.388922000	-0.222014000	3.239828000
8	0.244418000	1.884911000	0.131954000
6	-2.060289000	3.290801000	-1.030806000
1	-2.489809000	2.981647000	-1.982044000
6	-0.687965000	3.481359000	-0.954919000
1	-0.302026000	3.994793000	-0.077941000
6	-2.971524000	3.450173000	0.133583000
1	-3.839066000	4.076162000	-0.120206000
1	-3.380235000	2.472192000	0.437529000
1	-2.452884000	3.883558000	0.995052000
6	0.203946000	3.417729000	-2.156323000
1	0.286281000	4.421210000	-2.604073000
1	1.210396000	3.099759000	-1.872990000
1	-0.187486000	2.737800000	-2.918836000

$^{4}TS_{5}$:

26	-0.095086000	0.025326000	-0.228363000
7	-0.082866000	0.035401000	1.785268000
7	1.922964000	-0.009033000	-0.198591000
7	-2.034686000	-0.498570000	-0.174724000
7	-0.032441000	-0.554312000	-2.158156000
6	-1.195540000	0.035281000	2.628656000
6	2.772812000	-0.055497000	-1.290515000
6	1.000704000	0.266297000	2.618674000
6	2.752603000	0.232324000	0.898113000
6	-2.889187000	-0.430102000	0.913043000
6	1.080823000	-0.533896000	-3.005032000
6	-2.860267000	-0.795064000	-1.265901000
6	-1.105668000	-0.840121000	-2.982893000
6	-0.789338000	0.257643000	3.995364000
6	4.140025000	0.149312000	-0.882199000
6	0.569527000	0.398064000	3.989290000
6	4.128283000	0.325317000	0.473816000
6	-4.251007000	-0.692516000	0.510388000
6	0.681594000	-0.821948000	-4.362038000
6	-4.232738000	-0.919901000	-0.835231000
6	-0.670829000	-1.013586000	-4.347708000
1	-1.462487000	0.289129000	4.839029000
1	4.988605000	0.146289000	-1.549757000
1	1.229301000	0.567772000	4.826983000
1	4.965215000	0.496095000	1.134343000
1	-5.096939000	-0.708585000	1.181205000
1	1.357183000	-0.876191000	-5.202598000
1	-5.060520000	-1.157849000	-1.486384000
1	-1.322261000	-1.255465000	-5.174041000
6	-2.498676000	-0.168876000	2.222177000
6	2.377538000	-0.292536000	-2.605449000
6	-2.430401000	-0.951143000	-2.565380000
6	2.325215000	0.358982000	2.204778000
1	-3.273177000	-0.146767000	2.980861000
1	3.151046000	-0.306283000	-3.365300000
1	-3.174522000	-1.183478000	-3.319171000
1	3.076195000	0.539786000	2.965911000
8	-0.307058000	1.637158000	-0.472351000
6	-0.681154000	3.745909000	-0.944084000
1	-0.798295000	3.242079000	-1.895867000
6	-1.812299000	4.191239000	-0.292766000
1	-2.788682000	3.904244000	-0.678081000
6	-1.779791000	5.040277000	0.938848000
1	-2.197926000	6.031981000	0.687544000
1	-2.475479000	4.629966000	1.686932000
6	0.659583000	5.442402000	0.398419000
1	1.659550000	5.608898000	0.814106000
1	0.387991000	6.357654000	-0.145988000
6	-0.362267000	5.198115000	1.523568000
1	-0.347427000	6.021237000	2.246106000
1	-0.089985000	4.285046000	2.071426000
6	0.696037000	4.247455000	-0.579902000
1	1.263849000	3.417920000	-0.136926000
1	1.231493000	4.517687000	-1.500278000

$^{4}TS_{6}$:

26	0.099785000	0.155879000	-0.077344000
7	0.090790000	-0.035788000	1.921703000
7	2.056810000	-0.248752000	-0.065219000
7	-1.892432000	-0.007968000	-0.057824000
7	0.069065000	-0.249628000	-2.042772000
6	-1.006839000	0.096045000	2.773574000
6	2.903002000	-0.327559000	-1.174588000
6	1.198085000	-0.076483000	2.768506000
6	2.911675000	-0.248741000	1.038403000
6	-2.738089000	0.118240000	1.051286000
6	1.171162000	-0.346952000	-2.895977000
6	-2.754153000	-0.076845000	-1.159124000
6	-1.042716000	-0.288926000	-2.888557000
6	-0.577621000	0.116347000	4.150011000
6	4.278608000	-0.389406000	-0.753613000
6	0.782309000	0.007272000	4.147051000
6	4.283964000	-0.343702000	0.611526000
6	-4.114424000	0.134119000	0.633128000
6	0.739109000	-0.454418000	-4.266115000
6	-4.125072000	0.009077000	-0.728774000
6	-0.625992000	-0.423231000	-4.261492000
1	-1.242469000	0.195861000	4.997059000
1	5.120768000	-0.465774000	-1.424976000
1	1.455425000	-0.018134000	4.990874000
1	5.131411000	-0.374027000	1.279880000
1	-4.957023000	0.207358000	1.304647000
1	1.404277000	-0.553596000	-5.110843000
1	-4.977904000	-0.038515000	-1.389699000
1	-1.300842000	-0.490881000	-5.101739000
6	-2.324219000	0.178000000	2.368652000
6	2.491942000	-0.368159000	-2.492312000
6	-2.358703000	-0.210782000	-2.476459000
6	2.512839000	-0.175671000	2.358865000
1	-3.085785000	0.266000000	3.135207000
1	3.254776000	-0.444513000	-3.258612000
1	-3.131734000	-0.264295000	-3.234652000
1	3.281956000	-0.196474000	3.122264000
8	0.292455000	1.866164000	-0.150339000
6	-0.717860000	3.378642000	-1.008143000
1	-0.560551000	2.897016000	-1.967485000
6	-2.044602000	3.476517000	-0.541635000
1	-2.819473000	2.875870000	-1.009518000
6	-2.417828000	4.304088000	0.636501000
1	-3.285650000	4.937546000	0.375952000
1	-2.824094000	3.629708000	1.416038000
6	-0.083035000	5.197585000	0.631530000
1	0.689437000	5.831422000	1.057191000

6	-1.296520000	5.139453000	1.196929000
1	-1.518675000	5.716896000	2.089284000
6	0.285995000	4.431800000	-0.612363000
1	1.270050000	3.958790000	-0.506634000
1	0.396044000	5.129628000	-1.463651000

$^{4}TS_{7}$:

26	0.236500000	-0.184862000	0.000641000
7	-0.741483000	-0.887138000	1.608795000
7	0.177607000	-2.035009000	-0.790574000
7	-0.253644000	1.683564000	0.571896000
7	0.671858000	0.537254000	-1.829204000
6	-1.182956000	-0.157902000	2.712259000
6	0.646127000	-2.429250000	-2.042325000
6	-0.930202000	-2.223233000	1.961211000
6	-0.127294000	-3.220723000	-0.129181000
6	-0.755452000	2.079929000	1.807363000
6	1.077169000	-0.190233000	-2.943639000
6	0.054432000	2.869887000	-0.090345000
6	0.860219000	1.870037000	-2.178967000
6	-1.654303000	-1.045509000	3.747251000
6	0.619645000	-3.866326000	-2.159726000
6	-1.498115000	-2.320203000	3.283720000
6	0.143136000	-4.355786000	-0.976471000
6	-0.773378000	3.518846000	1.907930000
6	1.504874000	0.696416000	-3.996609000
6	-0.272276000	4.007164000	0.734941000
6	1.373221000	1.971821000	-3.522151000
1	-2.059272000	-0.721718000	4.694435000
1	0.919077000	-4.415610000	-3.039901000
1	-1.749768000	-3.247748000	3.775986000
1	-0.024990000	-5.384825000	-0.695800000
1	-1.132750000	4.069473000	2.764460000
1	1.851074000	0.375016000	-4.967575000
1	-0.139866000	5.037407000	0.439876000
1	1.589778000	2.900865000	-4.028053000
6	-1.184818000	1.222235000	2.806412000
6	1.068161000	-1.571810000	-3.042570000
6	0.580650000	2.957027000	-1.366557000
6	-0.636203000	-3.308711000	1.156328000
1	-1.559826000	1.664076000	3.722920000
1	1.405372000	-2.012005000	-3.974415000
1	0.771266000	3.946707000	-1.766516000
1	-0.835795000	-4.298254000	1.552254000
8	1.760269000	-0.234461000	0.629232000
6	3.309630000	-0.795116000	2.066954000
1	3.383151000	-1.762512000	1.586816000
1	2.578571000	-0.709441000	2.859719000
6	4.238989000	0.205713000	1.867721000
1	4.138870000	1.108394000	2.466166000
6	5.335979000	0.189039000	0.937886000
6	5.568698000	-0.881908000	0.027835000
6	6.656865000	-0.850327000	-0.834198000
6	7.543080000	0.242968000	-0.817697000
6	7.328992000	1.312751000	0.067804000
6	6.240470000	1.288587000	0.931399000
1	4.886696000	-1.723685000	-0.000635000
1	6.826629000	-1.670931000	-1.522336000
1	8.393653000	0.259114000	-1.490558000
1	8.013159000	2.153669000	0.077818000
1	6.072571000	2.111204000	1.619440000

$^{4}TS_{8}$:

26	0.018578000	1.709090000	0.226303000
7	-0.610557000	1.343118000	2.085493000
7	1.907008000	1.689746000	0.883430000
7	-1.812382000	1.269642000	-0.454751000
7	0.692539000	1.705142000	-1.651319000
6	-1.930909000	1.162045000	2.511573000
6	3.062638000	1.901555000	0.127085000
6	0.157841000	1.321572000	3.254331000
6	2.343935000	1.615074000	2.208403000
6	-2.973415000	1.083390000	0.304170000
6	2.008471000	1.923263000	-2.074755000
6	-2.229519000	1.186533000	-1.788075000
6	-0.055427000	1.584341000	-2.827930000
6	-1.976859000	1.046532000	3.944703000
6	4.216437000	1.946988000	0.986926000
6	-0.690715000	1.139272000	4.401050000
6	3.774461000	1.765303000	2.267763000
6	-4.105233000	0.887934000	-0.562209000
6	2.066137000	1.960636000	-3.511247000
6	-3.646456000	0.944616000	-1.849453000
6	0.797133000	1.745801000	-3.974893000
1	-2.880050000	0.896322000	4.517231000
1	5.229756000	2.091244000	0.642895000
1	-0.337091000	1.083647000	5.419750000
1	4.354867000	1.733754000	3.177745000
1	-5.114902000	0.716607000	-0.219727000
1	2.969283000	2.113941000	-4.082920000
1	-4.208140000	0.831608000	-2.764780000
1	0.460430000	1.691131000	-4.999433000
6	-3.034568000	1.059633000	1.685096000
6	3.110414000	2.032837000	-1.247828000
6	-1.414264000	1.338097000	-2.894386000
6	1.532739000	1.445221000	3.314024000
1	-4.002895000	0.908886000	2.147768000
1	4.077135000	2.195890000	-1.709461000
1	-1.867924000	1.254651000	-3.874990000
1	2.003979000	1.407312000	4.288943000
8	-0.185553000	3.791294000	0.396457000
6	-1.635064000	5.068033000	-1.340782000
6	-0.643708000	5.113641000	-0.201829000
6	-1.014363000	4.788005000	1.205115000
1	-2.028299000	4.423228000	1.352266000
1	0.214915000	5.768014000	-0.326503000
1	-1.326821000	5.737659000	-2.147848000
1	-2.635073000	5.369616000	-1.011398000
1	-1.707450000	4.057043000	-1.751137000
6	-0.371120000	5.382202000	2.398520000
6	-1.168995000	5.689754000	3.514183000
6	-0.604859000	6.296084000	4.640636000
6	0.763956000	6.585548000	4.667057000
6	1.568108000	6.266061000	3.564166000
6	1.005699000	5.669642000	2.433787000
1	1.634826000	5.406706000	1.589604000
1	2.630499000	6.484335000	3.586292000
1	1.202937000	7.055065000	5.540841000
1	-1.229966000	6.538909000	5.493036000
1	-2.231379000	5.463289000	3.497349000

$^{4}TS_{9}$:

26	0.213038000	0.048391000	-0.073393000
7	0.103017000	0.076869000	1.939440000
7	2.204491000	-0.189547000	0.083507000
7	-1.771163000	-0.310364000	-0.129753000
7	0.334892000	-0.576549000	-1.984732000
6	-1.049228000	0.195603000	2.712206000
6	3.116195000	-0.339809000	-0.960981000
6	1.157077000	0.215969000	2.838741000
6	2.988701000	-0.018832000	1.223768000
6	-2.680802000	-0.138408000	0.910381000
6	1.484798000	-0.674970000	-2.761853000
6	-2.550952000	-0.535689000	-1.258468000
6	-0.720434000	-0.771803000	-2.875155000
6	-0.708236000	0.401208000	4.097928000
6	4.468560000	-0.269131000	-0.464154000
6	0.656216000	0.411044000	4.176743000
6	4.389821000	-0.073862000	0.885105000
6	-4.031905000	-0.270932000	0.425199000
6	1.144314000	-0.944754000	-4.137620000
6	-3.951472000	-0.518994000	-0.916848000

6	-0.218199000	-1.006678000	-4.207153000
1	-1.426070000	0.509686000	4.897234000
1	5.352571000	-0.370450000	-1.075856000
1	1.275735000	0.530066000	5.053078000
1	5.196599000	0.017305000	1.596991000
1	-4.916007000	-0.193017000	1.040178000
1	1.862681000	-1.078148000	-4.932641000
1	-4.756692000	-0.683835000	-1.617111000
1	-0.836916000	-1.200143000	-5.070692000
6	-2.345020000	0.104826000	2.231288000
6	2.780335000	-0.554704000	-2.285802000
6	-2.061111000	-0.751410000	-2.537732000
6	2.500739000	0.169697000	2.504960000
1	-3.154747000	0.213052000	2.944426000
1	3.590130000	-0.659356000	-2.999394000
1	-2.782010000	-0.922686000	-3.329518000
1	3.222266000	0.282637000	3.306621000
8	0.181806000	1.694823000	-0.358365000
6	-0.001679000	3.897189000	-1.416125000
1	0.319426000	3.595831000	-2.410832000
6	0.913769000	3.728680000	-0.382513000
1	1.910306000	3.395528000	-0.658677000
6	-1.389174000	4.418896000	-1.304098000
1	-1.520758000	5.293563000	-1.957962000
1	-2.103067000	3.664112000	-1.663181000
1	-1.669433000	4.702678000	-0.288705000
6	0.734531000	4.158046000	1.035657000
1	1.198668000	3.433720000	1.711157000
1	1.241081000	5.121300000	1.201091000
1	-0.313227000	4.273825000	1.318166000

^{4}TS$_{10}$:

26	1.653062000	4.027115000	-1.076666000
7	1.208600000	2.079370000	-0.820475000
7	3.605118000	3.547461000	-1.012361000
7	-0.243995000	4.333820000	-1.690118000
7	2.157314000	5.797895000	-1.896558000
6	-0.055451000	1.499025000	-0.805538000
6	4.688740000	4.406489000	-1.186319000
6	2.073776000	1.071683000	-0.401322000
6	4.159422000	2.353841000	-0.563670000
6	-1.321938000	3.461587000	-1.558070000
6	3.425415000	6.362270000	-1.961114000
6	-0.798994000	5.531928000	-2.123887000
6	1.290365000	6.807319000	-2.310444000
6	0.026101000	0.120439000	-0.388657000
6	5.921766000	3.735720000	-0.852478000
6	1.342561000	-0.143274000	-0.136640000
6	5.594367000	2.467428000	-0.465825000
6	-2.551390000	4.122216000	-1.921431000
6	3.350526000	7.723930000	-2.431260000
6	-2.227993000	5.403935000	-2.269725000
6	2.029768000	7.999745000	-2.645841000
1	-0.818134000	-0.548181000	-0.310311000
1	6.901626000	4.184721000	-0.917496000
1	1.790004000	-1.070614000	0.188542000
1	6.252984000	1.671439000	-0.151947000
1	-3.524600000	3.654345000	-1.916983000
1	4.203014000	8.369489000	-2.581142000
1	-2.884192000	6.191995000	-2.607967000
1	1.586670000	8.915768000	-3.007233000
6	-1.234231000	2.143264000	-1.145834000
6	4.605338000	5.714999000	-1.625932000
6	-0.084563000	6.687644000	-2.404870000
6	3.445303000	1.202740000	-0.270882000
1	-2.153342000	1.570184000	-1.093345000
1	5.529991000	6.272949000	-1.723969000
1	-0.642853000	7.552658000	-2.745696000
1	4.004053000	0.336610000	0.066024000
8	1.489572000	4.521385000	0.480403000
6	0.837693000	9.481293000	3.991842000
6	0.294962000	8.045647000	3.901074000
6	0.892874000	7.284275000	2.587405000
6	0.452497000	7.359697000	5.261848000
6	-0.291140000	7.945827000	1.798058000
6	-1.085696000	7.959852000	3.143576000
6	-1.749593000	6.583652000	3.344571000
6	-0.817942000	5.431220000	2.849854000
6	0.636447000	5.831422000	2.745697000
6	1.650593000	4.896117000	2.766965000
1	2.689011000	5.191885000	2.674629000
1	1.450139000	3.846814000	2.948588000
1	-0.927757000	4.530741000	3.465167000
1	-1.123444000	5.131176000	1.834617000
1	-0.707279000	7.367162000	0.968856000
1	-2.000014000	6.439652000	4.401618000
1	-2.694656000	6.547262000	2.790914000
1	-1.789665000	8.778433000	3.320956000
1	-0.029297000	8.942391000	1.442306000
1	-0.119657000	7.904872000	6.022826000
1	0.111974000	6.320843000	5.268921000
1	1.503595000	7.367080000	5.575104000
1	1.923168000	7.518645000	2.311075000
1	0.761890000	10.032803000	3.050903000
1	0.271974000	10.038987000	4.748762000
1	1.890011000	9.478115000	4.299165000

UB3LYP/BS2 optimized geometries of epoxidation product complexes for the reaction of $[Fe^{IV}(O)(Por^{+\bullet})]^{+}$ with substrates:

^{4}P$_{1}$:

26	-0.067740000	0.151886000	0.060909000
7	-0.054528000	-0.014707000	2.038142000
7	1.921014000	0.168785000	0.054540000
7	-2.034597000	-0.137921000	0.054616000
7	-0.055685000	-0.003909000	-1.932264000
6	-1.159406000	-0.113774000	2.887985000
6	2.770010000	0.226434000	-1.055880000
6	1.052246000	0.062949000	2.887769000
6	2.769499000	0.232490000	1.164493000
6	-2.882232000	-0.210975000	1.164748000
6	1.054809000	0.045612000	-2.783997000
6	-2.882266000	-0.215550000	-1.055690000
6	-1.160123000	-0.129678000	-2.783982000
6	-0.732950000	-0.108671000	4.261225000
6	4.138517000	0.335307000	-0.629769000
6	0.630579000	0.000776000	4.261108000
6	4.137967000	0.338332000	0.738835000
6	-4.250178000	-0.323418000	0.739290000
6	0.634347000	-0.045031000	-4.154602000
6	-4.250552000	-0.325521000	-0.629335000
6	-0.730543000	-0.153369000	-4.154580000
1	-1.401659000	-0.180028000	5.106343000
1	4.982608000	0.391665000	-1.301384000
1	1.302258000	0.036885000	5.106120000
1	4.981408000	0.398908000	1.410778000
1	-5.092325000	-0.398477000	1.411388000
1	1.306778000	-0.033697000	-4.999863000
1	-5.092970000	-0.403898000	-1.300830000
1	-1.396398000	-0.248221000	-4.999795000
6	-2.477449000	-0.197183000	2.485044000
6	2.369643000	0.172677000	-2.377401000
6	-2.478623000	-0.208620000	-2.377250000
6	2.367147000	0.186470000	2.484820000
1	-3.240268000	-0.265408000	3.251455000
1	3.136163000	0.212966000	-3.142257000
1	-3.242159000	-0.290367000	-3.141794000
1	3.130947000	0.241045000	3.251347000
8	-0.224748000	2.242459000	0.035010000
6	-1.015359000	2.987626000	-1.020969000
1	-1.625849000	3.778131000	-0.598391000
1	-1.490089000	2.328503000	-1.740282000

6	0.459245000	3.101928000	-1.008521000
1	1.042766000	2.525260000	-1.718647000
1	0.932872000	3.976666000	-0.576390000

$^4P_2$:

26	-0.106443000	0.193080000	0.023100000
7	-0.061761000	-0.067075000	1.972031000
7	1.870592000	0.378281000	-0.006229000
7	-2.057006000	-0.192443000	0.031641000
7	-0.100493000	0.129450000	-1.951223000
6	-1.087016000	-0.516957000	2.803305000
6	2.727632000	0.274855000	-1.095269000
6	0.985851000	0.306774000	2.817076000
6	2.665703000	0.715299000	1.085010000
6	-2.835468000	-0.619330000	1.099990000
6	1.000926000	0.039237000	-2.806949000
6	-2.928216000	-0.020427000	-1.039867000
6	-1.223633000	0.236658000	-2.778991000
6	-0.653741000	-0.463142000	4.174463000
6	4.078685000	0.560553000	-0.676391000
6	0.606993000	0.073250000	4.184676000
6	4.036130000	0.862899000	0.657019000
6	-4.216109000	-0.714950000	0.686213000
6	0.553080000	0.075200000	-4.172657000
6	-4.277493000	-0.318234000	-0.620043000
6	-0.809292000	0.228124000	-4.155113000
1	-1.256443000	-0.770907000	5.016251000
1	4.936768000	0.541014000	-1.332147000
1	1.241023000	0.275405000	5.035406000
1	4.855062000	1.120304000	1.312579000
1	-5.023345000	-1.033077000	1.329423000
1	1.207155000	0.011863000	-5.029927000
1	-5.142275000	-0.264311000	-1.264883000
1	-1.484585000	0.298301000	-4.995237000
6	-2.374318000	-0.817751000	2.390173000
6	2.325836000	0.066346000	-2.403597000
6	-2.540874000	0.231207000	-2.345484000
6	2.237402000	0.732483000	2.402237000
1	-3.084741000	-1.144112000	3.140267000
1	3.090896000	0.010155000	-3.168737000
1	-3.318140000	0.317233000	-3.095798000
1	2.960764000	0.990986000	3.166630000
8	-0.474788000	2.267579000	0.188132000
6	-1.161865000	3.192903000	-0.777679000
1	-2.028165000	3.689968000	-0.353512000
1	-1.284615000	2.763696000	-1.767960000
6	0.206859000	3.529427000	-0.314957000
1	0.298041000	4.253222000	0.490828000
6	1.408914000	3.346345000	-1.196093000
1	2.284579000	3.051307000	-0.608886000
1	1.223327000	2.582594000	-1.955747000
1	1.634650000	4.296209000	-1.697746000

$^4P_3$:

26	-0.020864000	0.309358000	0.050850000
7	-0.021844000	0.002196000	2.015812000
7	1.962932000	0.379477000	0.073768000
7	-1.976304000	-0.062453000	0.006550000
7	0.023019000	0.215493000	-1.937925000
6	-1.118126000	-0.270121000	2.836166000
6	2.833667000	0.408293000	-1.019771000
6	1.057034000	0.162544000	2.887493000
6	2.783588000	0.503262000	1.198673000
6	-2.820744000	-0.318609000	1.089890000
6	1.145140000	0.244844000	-2.771545000
6	-2.817264000	-0.006603000	-1.108622000
6	-1.076469000	0.236943000	-2.802606000
6	-0.710504000	-0.301346000	4.216010000
6	4.189474000	0.566882000	-0.569167000
6	0.628032000	-0.022349000	4.248502000
6	4.157095000	0.638913000	0.797606000
6	-4.183714000	-0.418611000	0.643097000
6	0.737901000	0.278928000	-4.148905000
6	-4.183501000	-0.212529000	-0.710156000
6	-0.631408000	0.290936000	-4.168062000
1	-1.375699000	-0.502575000	5.042812000
1	5.047585000	0.612107000	-1.223668000
1	1.279740000	0.046458000	5.107062000
1	4.983683000	0.750798000	1.483807000
1	-5.023707000	-0.620379000	1.291448000
1	1.420845000	0.296933000	-4.985591000
1	-5.022706000	-0.217522000	-1.390265000
1	-1.290665000	0.313113000	-5.023472000
6	-2.420984000	-0.433881000	2.407406000
6	2.456999000	0.321534000	-2.346110000
6	-2.402442000	0.168667000	-2.415496000
6	2.359209000	0.429125000	2.511680000
1	-3.179936000	-0.638897000	3.153071000
1	3.235832000	0.346077000	-3.099075000
1	-3.159775000	0.193786000	-3.190229000
1	3.102267000	0.535993000	3.293027000
8	-0.331923000	2.351731000	0.221697000
6	-1.391112000	3.191508000	-0.446938000
1	-2.064331000	3.663255000	0.261147000
1	-1.848977000	2.697315000	-1.299833000
6	0.025438000	3.616067000	-0.552922000
1	0.375355000	4.382555000	0.134792000
6	0.810538000	3.459691000	-1.827041000
1	1.811583000	3.079066000	-1.590362000
1	0.322820000	2.722485000	-2.471314000
6	0.915232000	4.817984000	-2.551951000
1	1.418204000	5.564412000	-1.925119000
1	1.491937000	4.707713000	-3.475872000
1	-0.077181000	5.203632000	-2.815011000

$^4P_4$:

26	-0.019668000	0.158169000	-0.019994000
7	0.036529000	-0.096300000	1.951764000
7	1.964686000	0.217481000	-0.059125000
7	-1.981866000	-0.194055000	-0.001823000
7	-0.035066000	0.017513000	-2.012671000
6	-1.044588000	-0.299317000	2.811462000
6	2.799629000	0.246581000	-1.180450000
6	1.147004000	0.040923000	2.785832000
6	2.822224000	0.343920000	1.038529000
6	-2.801746000	-0.343679000	1.118136000
6	1.062900000	0.030204000	-2.879507000
6	-2.851566000	-0.176400000	-1.094572000
6	-1.156931000	-0.046631000	-2.847094000
6	-0.595319000	-0.316853000	4.178557000
6	4.168349000	0.414329000	-0.775095000
6	0.754919000	-0.099354000	4.163208000
6	4.181303000	0.484579000	0.592018000
6	-4.181345000	-0.410207000	0.717485000
6	0.618315000	-0.028628000	-4.245111000
6	-4.213023000	-0.298703000	-0.646645000
6	-0.749871000	-0.065629000	-4.225534000
1	-1.242261000	-0.468413000	5.030064000
1	5.003893000	0.462977000	-1.457942000
1	1.436028000	-0.040028000	4.999372000
1	5.029803000	0.599034000	1.250516000
1	-5.008889000	-0.532108000	1.400861000
1	1.278005000	-0.038585000	-5.100364000
1	-5.071330000	-0.315941000	-1.302301000
1	-1.432343000	-0.115628000	-5.061322000
6	-2.366425000	-0.413708000	2.427665000
6	2.385036000	0.138631000	-2.493524000
6	-2.470854000	-0.098129000	-2.421009000
6	2.441781000	0.275755000	2.364871000
1	-3.110391000	-0.550142000	3.203594000

1	3.141781000	0.155092000	-3.268855000
1	-3.248975000	-0.119630000	-3.174936000
1	3.211962000	0.376215000	3.120397000
8	-0.405152000	2.174989000	0.123593000
6	-1.260248000	2.958305000	-0.865289000
1	-1.325155000	2.434759000	-1.816802000
6	0.078789000	3.482278000	-0.496673000
1	0.111361000	4.256298000	0.266907000
6	-2.543244000	3.473751000	-0.286271000
1	-2.986741000	4.215197000	-0.963015000
1	-3.257226000	2.651761000	-0.160216000
1	-2.369027000	3.943991000	0.686685000
6	1.263892000	3.380275000	-1.411189000
1	1.394675000	4.330431000	-1.944620000
1	2.177556000	3.179908000	-0.841165000
1	1.125437000	2.580325000	-2.142707000

$^4P_5$:

26	-0.044483000	0.057668000	0.034777000
7	-0.170289000	-0.073875000	2.019722000
7	1.921134000	-0.017024000	0.162487000
7	-2.009163000	-0.185213000	-0.106343000
7	0.076563000	0.034098000	-1.959948000
6	-1.327673000	0.049876000	2.782883000
6	2.822770000	0.257972000	-0.869537000
6	0.869431000	-0.254291000	2.924715000
6	2.697878000	-0.218405000	1.305109000
6	-2.933371000	-0.084777000	0.939915000
6	1.211664000	0.324103000	-2.709545000
6	-2.765152000	-0.414808000	-1.260903000
6	-0.943939000	-0.191209000	-2.877214000
6	-0.995288000	-0.020657000	4.186283000
6	4.166815000	0.209156000	-0.362038000
6	0.351433000	-0.232052000	4.272377000
6	4.091465000	-0.109711000	0.968007000
6	-4.264245000	-0.256389000	0.427620000
6	0.884198000	0.305659000	-4.115416000
6	-4.159183000	-0.483102000	-0.920166000
6	-0.435139000	-0.034505000	-4.219236000
1	-1.717385000	0.047634000	4.986598000
1	5.049509000	0.381569000	-0.960117000
1	0.956743000	-0.356049000	5.158220000
1	4.899619000	-0.236872000	1.673163000
1	-5.158212000	-0.226406000	1.033125000
1	1.593027000	0.496334000	-4.907757000
1	-4.951434000	-0.662838000	-1.632036000
1	-1.026919000	-0.164780000	-5.113347000
6	-2.615762000	0.082490000	2.278351000
6	2.488715000	0.468432000	-2.196259000
6	-2.265735000	-0.440755000	-2.552217000
6	2.207559000	-0.362864000	2.591050000
1	-3.434342000	0.150968000	2.985125000
1	3.293905000	0.673696000	-2.891730000
1	-2.966227000	-0.604040000	-3.362496000
1	2.922265000	-0.498432000	3.393902000
8	-0.147304000	2.144939000	0.011506000
6	-1.052684000	2.902284000	-0.957398000
1	-1.699740000	2.212522000	-1.492593000
6	-1.261647000	3.047187000	0.509822000
1	-2.065300000	2.476828000	0.968519000
6	-0.776564000	4.273607000	1.240916000
1	-1.643390000	4.938322000	1.374007000
1	-0.431601000	3.984833000	2.241823000
6	-0.114663000	5.275916000	-0.989353000
1	0.626873000	5.887550000	-1.514731000
1	-1.052900000	5.848937000	-0.973614000
6	0.330829000	5.014111000	0.462560000
1	0.555622000	5.960228000	0.966609000
1	1.244828000	4.406919000	0.468295000
6	-0.334699000	3.958953000	-1.769651000
1	0.630505000	3.536335000	-2.080824000
1	-0.909784000	4.150499000	-2.683851000

$^4P_6$:

26	-0.124772000	0.120058000	-0.025232000
7	-0.236421000	-0.029069000	1.961605000
7	1.866203000	0.123653000	0.100083000
7	-2.081957000	-0.293613000	-0.144871000
7	0.016777000	-0.083310000	-2.006839000
6	-1.389173000	-0.172910000	2.742255000
6	2.781624000	0.175718000	-0.954571000
6	0.814031000	0.091582000	2.877877000
6	2.638641000	0.221163000	1.260774000
6	-2.990658000	-0.420344000	0.913384000
6	1.176751000	-0.003215000	-2.785726000
6	-2.851150000	-0.437075000	-1.306341000
6	-1.029765000	-0.239416000	-2.923545000
6	-1.048905000	-0.129225000	4.139191000
6	4.121799000	0.294720000	-0.442722000
6	0.306695000	0.034938000	4.222522000
6	4.033615000	0.323650000	0.920996000
6	-4.317378000	-0.638611000	0.402668000
6	0.843095000	-0.096760000	-4.181553000
6	-4.231691000	-0.647920000	-0.962751000
6	-0.514586000	-0.241638000	-4.266552000
1	-1.761241000	-0.224170000	4.945206000
1	5.008325000	0.346527000	-1.056917000
1	0.918368000	0.100358000	5.109961000
1	4.833827000	0.403431000	1.641519000
1	-5.194540000	-0.774873000	1.017560000
1	1.563366000	-0.066178000	-4.985473000
1	-5.025079000	-0.793416000	-1.680706000
1	-1.120230000	-0.353089000	-5.153598000
6	-2.671742000	-0.349702000	2.257070000
6	2.463790000	0.124510000	-2.298309000
6	-2.365304000	-0.394419000	-2.600341000
6	2.151740000	0.217699000	2.553754000
1	-3.475446000	-0.459640000	2.975642000
1	3.275589000	0.172655000	-3.014426000
1	-3.072610000	-0.516869000	-3.412202000
1	2.865006000	0.300236000	3.365268000
8	-0.297473000	2.207405000	-0.049949000
6	-1.100243000	3.156628000	-0.934245000
1	-1.794517000	2.614166000	-1.569391000
6	-1.343708000	3.155010000	0.529448000
1	-2.206914000	2.612708000	0.903704000
6	-0.782273000	4.236912000	1.413604000
1	-1.632263000	4.817877000	1.806217000
1	-0.313115000	3.772409000	2.292508000
6	0.410155000	5.157851000	-0.606522000
1	1.118830000	5.861072000	-1.034877000
6	0.190766000	5.155956000	0.714019000
1	0.722394000	5.857689000	1.350529000
6	-0.283770000	4.241429000	-1.586113000
1	0.444726000	3.779649000	-2.267524000
1	-0.961755000	4.823905000	-2.230468000

$^4P_7$:

26	0.701657000	-0.156985000	-0.432521000
7	0.878918000	-1.435616000	1.072526000
7	0.052221000	-1.633920000	-1.592092000
7	0.977771000	1.350938000	0.838736000
7	0.220611000	1.155173000	-1.849027000
6	1.125505000	-1.148316000	2.417266000
6	-0.447297000	-1.539497000	-2.892255000
6	0.877874000	-2.830360000	0.988517000
6	0.144070000	-3.001684000	-1.331133000
6	1.175020000	1.279727000	2.219252000
6	-0.290360000	0.885500000	-3.120366000
6	1.091398000	2.708475000	0.520314000
6	0.447939000	2.533757000	-1.827899000
6	1.285391000	-2.367177000	3.161885000

6	-0.688996000	-2.853910000	-3.426401000
6	1.150891000	-3.403622000	2.278994000
6	-0.309827000	-3.755426000	-2.469925000
6	1.406109000	2.592129000	2.754809000
6	-0.381753000	2.099903000	-3.884646000
6	1.376555000	3.471212000	1.705423000
6	0.088465000	3.113723000	-3.093714000
1	1.480056000	-2.409044000	4.223230000
1	-1.086379000	-3.047074000	-4.412006000
1	1.202203000	-4.464651000	2.475059000
1	-0.340223000	-4.834228000	-2.513670000
1	1.576617000	2.800780000	3.800715000
1	-0.755631000	2.156291000	-4.896427000
1	1.506077000	4.543573000	1.723297000
1	0.168930000	4.165259000	-3.327504000
6	1.230862000	0.115663000	2.960217000
6	-0.625676000	-0.366830000	-3.600245000
6	0.889389000	3.255442000	-0.733799000
6	0.567449000	-3.563440000	-0.141339000
1	1.417291000	0.198777000	4.023455000
1	-1.018367000	-0.436143000	-4.607794000
1	1.015038000	4.325785000	-0.848211000
1	0.596476000	-4.644350000	-0.070872000
8	2.630385000	-0.282806000	-1.166278000
6	3.585470000	0.855902000	-1.373920000
1	3.273659000	1.764298000	-0.867775000
1	3.898190000	0.968814000	-2.406712000
6	4.074333000	-0.286802000	-0.570397000
1	4.635530000	-1.053789000	-1.096261000
6	4.243078000	-0.260167000	0.895630000
6	4.297581000	0.945168000	1.615205000
6	4.524144000	0.928988000	2.994346000
6	4.685490000	-0.289845000	3.665743000
6	4.620084000	-1.497009000	2.954908000
6	4.408236000	-1.481582000	1.574995000
1	4.171394000	1.892945000	1.104049000
1	4.577007000	1.864228000	3.541040000
1	4.871172000	-0.299655000	4.734809000
1	4.744741000	-2.441835000	3.472278000
1	4.367277000	-2.412762000	1.017955000

$^4P_8$:

26	0.087886000	2.028359000	0.178293000
7	-0.298982000	1.484909000	2.059669000
7	2.043262000	1.834077000	0.540101000
7	-1.853899000	1.810823000	-0.258409000
7	0.487581000	2.169031000	-1.774842000
6	-1.553831000	1.327219000	2.655785000
6	3.094675000	2.032206000	-0.359104000
6	0.623207000	1.307699000	3.095590000
6	2.655428000	1.610406000	1.776504000
6	-2.905690000	1.602642000	0.641178000
6	1.744443000	2.319681000	-2.372382000
6	-2.463284000	1.929411000	-1.512720000
6	-0.429855000	2.239384000	-2.829823000
6	-1.404510000	1.055954000	4.060951000
6	4.357411000	1.929327000	0.323945000
6	-0.064328000	1.041078000	4.330994000
6	4.086835000	1.666656000	1.638202000
6	-4.163039000	1.604900000	-0.058066000
6	1.599334000	2.496950000	-3.792315000
6	-3.890495000	1.802300000	-1.383903000
6	0.261588000	2.442711000	-4.074260000
1	-2.225854000	0.890428000	4.742049000
1	5.320524000	2.036844000	-0.152353000
1	0.425366000	0.862326000	5.276563000
1	4.785412000	1.517798000	2.448017000
1	-5.124094000	1.458329000	0.412067000
1	2.422204000	2.629127000	-4.478959000
1	-4.585410000	1.850152000	-2.209086000
1	-0.222121000	2.523970000	-5.036288000
6	-2.769048000	1.395127000	2.001054000
6	2.958326000	2.273535000	-1.712969000
6	-1.802930000	2.133375000	-2.709764000
6	1.997611000	1.368887000	2.967518000
1	-3.669633000	1.247343000	2.585339000
1	3.860148000	2.405430000	-2.299117000
1	-2.398068000	2.201468000	-3.612856000
1	2.597591000	1.208771000	3.855520000
8	0.043895000	4.075898000	0.634601000
6	-0.410363000	5.654772000	-1.355680000
6	0.006321000	5.486503000	0.075602000
6	-0.954378000	5.123321000	1.153020000
1	-1.964607000	4.890338000	0.825984000
1	0.895374000	6.031197000	0.384217000
1	0.390553000	5.350206000	-2.036299000
1	-0.628209000	6.712709000	-1.545935000
1	-1.305718000	5.070868000	-1.585694000
6	-0.820691000	5.533029000	2.567878000
6	-1.986795000	5.818188000	3.299613000
6	-1.900660000	6.257450000	4.624008000
6	-0.649030000	6.399758000	5.233196000
6	0.516952000	6.100982000	4.514682000
6	0.434536000	5.672189000	3.188574000
1	1.339688000	5.425689000	2.643097000
1	1.487349000	6.205165000	4.988167000
1	-0.580967000	6.739297000	6.261138000
1	-2.805233000	6.485468000	5.177238000
1	-2.959748000	5.706919000	2.829204000
